# Rel-dependent decrease in the expression of ribosomal protein genes by inhibition of the respiratory electron transport chain in *Mycobacterium smegmatis*

**DOI:** 10.3389/fmicb.2024.1448277

**Published:** 2024-08-12

**Authors:** Na-Kyeong Kim, Jong-Eun Baek, Ye-Jin Lee, Yuna Oh, Jeong-Il Oh

**Affiliations:** ^1^Department of Integrated Biological Science, Pusan National University, Busan, Republic of Korea; ^2^Microbiological Resource Research Institute, Pusan National University, Busan, Republic of Korea

**Keywords:** electron transport chain, MprBA two-component system, *Mycobacterium*, respiration, ribosome, SigB, SigE, stringent response

## Abstract

In this study, we demonstrated that both the expression of most ribosomal protein genes and the amount of ribosomes were decreased in the Δ*aa*_3_ mutant of *Mycobacterium smegmatis*, in which the major terminal oxidase (*aa*_3_ cytochrome *c* oxidase) of the respiratory electron transport chain (ETC) is inactivated, compared to those in the wild-type strain. Deletion of the *rel* gene encoding the major (p)ppGpp synthetase in the background of the Δ*aa*_3_ mutant restored the reduced expression of ribosomal protein genes, suggesting that inhibition of the respiratory ETC leads to the Rel-dependent stringent response (SR) in this bacterium. Both a decrease in the expression of ribosomal protein genes by overexpression of *rel* and the increased expression of *rel* in the Δ*aa*_3_ mutant relative to the wild-type strain support the Rel-dependent induction of SR in the Δ*aa*_3_ mutant. We also demonstrated that the expression of ribosomal protein genes was decreased in *M. smegmatis* exposed to respiration-inhibitory conditions, such as KCN and bedaquiline treatment, null mutation of the cytochrome *bcc*_1_ complex, and hypoxia. The MprBA-SigE-SigB regulatory pathway was implicated in both the increased expression of *rel* and the decreased expression of ribosomal protein genes in the Δ*aa*_3_ mutant of *M. smegmatis*.

## Introduction

Mycobacteria encounter a myriad of stress conditions, such as nutrient deprivation, hypoxia, and oxidative stress, when exposed to host environments (Bretl et al., 2011). The stringent response (SR) helps mycobacteria adapt to these hostile conditions by modulating their metabolism and redirecting resources toward functions that are beneficial to them under given conditions (Gupta et al., 2021). The SR is mediated by the secondary messenger molecules, guanosine tetraphosphate (ppGpp) and guanosine pentaphosphate (pppGpp) [collectively referred to as (p)ppGpp] (Bange et al., 2021). In mycobacteria, SR has been suggested to affect survival under nutrient starvation, oxidative stress, and hypoxic conditions, as well as persistence, virulence, biofilm formation, cell division, cell morphology, and antibiotic tolerance, etc. (Primm et al., 2000; Betts et al., 2002; Dahl et al., 2003, 2005; Stallings et al., 2009; Klinkenberg et al., 2010; Weiss and Stallings, 2013; Gupta et al., 2016; Dutta et al., 2019). (p)ppGpp is produced by (p)ppGpp synthetase from the reactants ATP and GTP (or GDP). Two classes of (p)ppGpp synthetase occur in mycobacteria: the long Rel/SpoT homolog (RSH) and the small alarmone synthetase (SAS) (Prusa et al., 2018). The *Rv2583c* and *MSMEG_2965* genes encode the Rel proteins of the long RSH class in *Mycobacterium tuberculosis* and *Mycobacterium smegmatis*, respectively. Rel is a bifunctional enzyme with both (p)ppGpp synthetase and hydrolase activities and consists of an N-terminal catalytic domain with hydrolase/synthetase activities and a C-terminal regulatory domain (Avarbock et al., 1999, 2000, 2005; Primm et al., 2000; Singal et al., 2017; Bange et al., 2021). The C-terminal domain of Rel in mycobacteria harbors two subdomains: the “ThrRS, GTPase, and SpoT” (TGS) domain and “Aspartate kinase, Chorismate mutase and TyrA” (ACT) domain (Jain et al., 2006; Prusa et al., 2018; Bange et al., 2021; Shin et al., 2021a,b). *M. tuberculosis* and *M. smegmatis* have a single copy of the gene encoding SAS (*Rv1366* in *M. tuberculosis* and *MSMEG_5849* in *M. smegmatis*) (Murdeshwar and Chatterji, 2012; Weiss and Stallings, 2013). The Rv1366 protein was suggested to be catalytically inactive, while MSMEG_5849 annotated as RelZ was shown to be catalytically active and synthesize pGpp using GMP and ATP in addition to (p)ppGpp (Weiss and Stallings, 2013; Bag et al., 2014; Petchiappan et al., 2020). RelZ of *M. smegmatis* has been demonstrated to be a dual-domain SAS with the RNase HII and (p)ppGpp (pGpp) synthetase activities (Murdeshwar and Chatterji, 2012; Krishnan et al., 2016; Krishnan and Chatterji, 2020; Petchiappan et al., 2020). The expression of *relZ* was shown to be upregulated in *M. smegmatis* exposed to replication stress, such as UV stress (Krishnan et al., 2016), and the pGpp synthetase activity of RelZ was demonstrated to be inhibited by single-stranded RNA and pppGpp (Petchiappan et al., 2020).

The most prominent phenotypes of SR induction in bacteria are the downregulation of rRNA and ribosomal protein genes, along with the upregulation of amino acid biosynthesis genes (Prusa et al., 2018; Bange et al., 2021). In SR-induced mycobacteria, the expression of rRNA and ribosomal protein genes has been shown to be downregulated in a Rel-dependent manner (Dahl et al., 2003, 2005; Stallings et al., 2009; Prusa et al., 2018). In contrast, a RelZ-dependent decrease in the expression of rRNA and ribosomal protein genes has not been reported in mycobacteria. Currently, the role of RelZ is not certain, but based on its RNase HII activity, it has been suggested that RelZ plays a role in removing the R-loop during UV-induced replication stress (Krishnan et al., 2016).

Mycobacteria have the bifurcate respiratory electron transport chain (ETC) that is terminated with two terminal oxidases (Kana et al., 2001; Matsoso et al., 2005). One branch is terminated with the *aa*_3_ cytochrome *c* oxidase, while the other is terminated with the *bd* quinol oxidase. The *aa*_3_ cytochrome *c* oxidase forms a supercomplex with the cytochrome *bcc*_1_ complex (Megehee et al., 2006; Wiseman et al., 2018; Zhou et al., 2021). Since the *aa*_3_ cytochrome *c* oxidase is the major terminal oxidase in *M. smegmatis* and *M. tuberculosis* grown aerobically, the *bcc*_1_-*aa*_3_ branch is required for optimal growth of the mycobacteria under aerobic conditions. Disruption of this branch results in growth impairment and upregulation of the *bd* quinol oxidase genes (Kana et al., 2001; Matsoso et al., 2005; Jeong et al., 2018; Beites et al., 2019; Ko and Oh, 2020). The *bd* quinol oxidase has been shown to have a high affinity for oxygen, thereby considered to play a crucial role under oxygen-limiting conditions and when the *aa*_3_ cytochrome *c* oxidase is inactivated (Puustinen et al., 1991; Kana et al., 2001; Belevich et al., 2007).

The MprBA two-component system (TCS), consisting of the membrane-bound MprB histidine kinase and its cognate MprA response regulator, was initially found to be necessary for *M. tuberculosis* to cause persistent infection (Zahrt and Deretic, 2001; Zahrt, 2003). In *M. tuberculosis* and *M. smegmatis*, the MprBA TCS has been found to be activated upon exposure to surface stress and alkaline pH (He et al., 2006; Pang et al., 2007; White et al., 2010). Besides ATP, inorganic polyphosphate synthesized by polyphosphate kinase was found to serve as a phosphate donor for MprB-mediated MprA phosphorylation (Sureka et al., 2007). The MprBA-SigE-Rel regulatory pathway has been suggested to be implicated in induction of (p)ppGpp synthesis and SR in mycobacteria (Sureka et al., 2007; Sanyal et al., 2013). In *M. tuberculosis* and *M. smegmatis*, the phosphorylated MprA positively regulates the expression of *sigE*, and the alternative sigma factor SigE in turn binds to the promoter of *rel* to activate its transcription (He et al., 2006; Sureka et al., 2007). Recently, we reported that the SigE-SigB regulatory pathway is activated in an *aa*_3_ oxidase mutant of *M. smegmatis* grown aerobically relative to the isogenic wild-type (WT) strain grown under the same conditions (Oh et al., 2022). This finding suggests the possibility that SR could be induced in *M. smegmatis* under respiration-inhibitory conditions. In this study, we demonstrate that SR is indeed induced in *M. smegmatis* through the MprBA-SigE-SigB-Rel signaling pathway when the respiratory ETC is inhibited.

## Materials and methods

### Bacterial strains, plasmids, and culture conditions

The bacterial strains and plasmids used in this study are listed in Table 1. *Escherichia coli* strains were grown in Lysogeny Broth (LB) medium at 37°C. *M. smegmatis* strains were grown in Middlebrook 7H9 medium (Difco, Sparks, MD, United States) supplemented with 0.2% (w/v) glucose (7H9-glucose) and 0.02% (v/v) Tween 80 as an anti-clumping agent at 37°C. For glucose-limiting conditions, *M. smegmatis* strains were grown aerobically in 7H9 medium supplemented with 0.01% (w/v) glucose and 0.02% (v/v) Tween 80. For hypoxic growth of *M. smegmatis* strains, a 100-ml flask was filled with 80 mL of aerobically grown culture with an optical density at 600 nm (OD_600_) of 0.7–0.75 and tightly sealed with a rubber stopper. The culture was incubated on a gyratory shaker for 3 h at 37°C, allowing for a gradual depletion of O_2_ from the growth medium. For treatment of *M. smegmatis* cultures with potassium cyanide (KCN), *M. smegmatis* strains were grown until the OD_600_ reached 0.45–0.5. Following the addition of KCN to the cultures to a final concentration of 50 μΜ, the cultures were further grown for 2 h. Ampicillin (100 μg/mL for *E. coli*), kanamycin (50 μg/mL for *E. coli* and 30 μg/mL for *M. smegmatis*), and hygromycin (200 μg/mL for *E. coli* and 50 μg/mL for *M. smegmatis*) were added to the growth medium when required.

**Table 1:** Strains and plasmids used in this study.

Strain/plasmid	Relevant phenotype/genotype^*^	Reference
Strains		
*E. coli* DH5α	φ80d*lacZ*ΔM15 Δ*lacU169 recA1 endA1 hsdR17 supE44 thi1 gyrA96 relA1*	Jessee (1986)
*M. smegmatis* mc^2^155	High-transformation-efficiency mutant of *M. smegmatis* ATCC 607	Snapper et al. (1990)
*M. smegmatis* Δ*sigE*	*MSMEG_5072* (*sigE*) deletion mutant of *M. smegmatis* mc^2^155	Oh et al. (2022)
*M. smegmatis* Δ*sigB*	*MSMEG_2752* (*sigB*) deletion mutant of *M. smegmatis* mc^2^155	Oh et al. (2022)
*M. smegmatis* Δ*aa*_3_	*MSMEG_4268* (*ctaC*) deletion mutant of *M. smegmatis* mc^2^155	Jeong et al. (2018)
*M. smegmatis* Δ*mprA*	*MSMEG_5488* (*mprA*) deletion mutant of *M. smegmatis* mc^2^155	This study
*M. smegmatis* Δ*rel*	*MSMEG_2965* (*rel*) deletion mutant of *M. smegmatis* mc^2^155	This study
*M. smegmatis* Δ*bc*_1_	*MSMEG_4263* (*qcrB*) deletion mutant of *M. smegmatis* mc^2^155	This study
*M. smegmatis* Δ*aa*_3_Δ*sigE*	*MSMEG_4268* (*ctaC*) and *MSMEG_5072* (*sigE*) double-deletion mutant of *M. smegmatis* mc^2^155	Oh et al. (2022)
*M. smegmatis* Δ*aa*_3_Δ*sigB*	*MSMEG_4268* (*ctaC*) and *MSMEG_2752* (*sigB*) double-deletion mutant of *M. smegmatis* mc^2^155	Oh et al. (2022)
*M. smegmatis* Δ*aa*_3_Δ*mprA*	*MSMEG_4268* (*ctaC*) and *MSMEG_5488* (*mprA*) double-deletion mutant of *M. smegmatis* mc^2^155	This study
*M. smegmatis* Δ*aa*_3_Δ*rel*	*MSMEG_4268* (*ctaC*) and *MSMEG_2965* (*rel*) double-deletion mutant of *M. smegmatis* mc^2^155	This study
Plasmids		
pNC	Hyg^r^; promoterless *lacZ*	Oh et al. (2010)
pNCII	Hyg^r^; a derivative of pNC, the ribosome-binding site of *lacZ* is removed (translational fusion)	Oh et al. (2020)
pMH201	Km^r^; acetamide-inducible promoter, derivative of pMV306	Kang et al. (2005)
pKOTs	Hyg^r^; pKO-based vector constructed by inserting HindIII-KpnI fragment containing pAL500Ts and the pUC ori derived from pDE	Jeong et al. (2013)
pMV306	Km^r^; integration vector containing the *int* and *attP* sites of mycobacteriophage L5 for integration into the mycobacterial genome	Stover et al. (1991)
pUC19	Amp^r^; l*acPOZ*’	Yanisch-Perron et al. (1985)
pNCIIrel	pNCII::0.436-kb XbaI-ClaI fragment containing the *MSMEG_2965* (*rel*) promoter region	This study
pNCIIeis	pNCII::0.508-kb XbaI-BamHI fragment containing the *Rv2416c* (*eis*) promoter region	This study
pNCIIwag31	pNCII::0.490-kb XbaI-ClaI fragment containing the *Rv2145c* (*wag31*) promoter region	This study
pUC19mprA	pUC19::1.472-kb HindIII-EcoRV fragment containing *mprA* of *M. smegmatis*	This study
pUC19ΔmprA	pUC19::1.337-kb HindIII-EcoRV fragment containing Δ*mprA*	This study
pKOTsΔmprA	pKOTs::1.337-kb HindIII-EcoRV fragment from pUC19Δ*mprA*	This study
pKOTsΔrel	pKOTs::0.736-kb NotI-HindIII fragment containing Δ*rel*	This study
pKOTsΔbc_1_	pKOTs::0.818-kb NotI-HindIII fragment containing Δ*bc*_1_	This study
pMH201rel	pMV306::2.417-kb NdeI-ClaI fragment containing C-terminally His-tagged *MSMEG_2965* (*rel*)	This study
pMV306ctaC	pMV306::1.37-kb XbaI-HindIII fragment containing *MSMEG_4268* (*ctaC*)	Jeong et al. (2018)

*Abbreviations: Amp^r^, ampicillin resistance; Hyg^r^, hygromycin resistance; Km^r^, kanamycin resistance.

### DNA manipulation and electroporation

Standard protocols and manufacturers’ instructions were followed for recombinant DNA manipulations (Green and Sambrook, 2012). Transformation of *M. smegmatis* with plasmids was carried out by electroporation as described elsewhere (Snapper et al., 1990). The primers used for PCR are listed in Table 2.

**Table 2:** Oligonucleotides used in this study.

Oligonucleotide	Nucleotide sequences (5′ → 3′)	Purpose
F_mprAmut	ATATAAGCTTGTGCAATCGTCGCCGTCG	Δ*mprA* construction
R_mprAmut	GTTCGCCGGAGATGACGTC	Δ*mprA* construction
F_relmut	ATTTGCGGCCGCCGTGCAGTCTCCTCCC	Δ*rel* construction
R_relmut	ATTTAAGCTTGCCGCATCCACGCCATGTC	Δ*rel* construction
F_relrec	CATGGACACCACCACGCTGGTCAAGGGCCGCGACTTCG	Δ*rel* construction
R_relrec	CGAAGTCGCGGCCCTTGACCAGCGTGGTGGTGTCCATG	Δ*rel* construction
F_bc1mut	ATTAGCGGCCGCGTCATCACCTTCCTCATGG	Δ*bc*_1_ construction
R_bc1mut	ATTAAAGCTTGGGAGTGGCGCATATAGAAAG	Δ*bc*_1_ construction
F_bc1rec	CTTCTGCTGCTGATCGGTTACGTCGAGCTGCACCAG	Δ*bc*_1_ construction
R_bc1rec	CTGGTGCAGCTCGACGTAACCGATCAGCAGCAGAAG	Δ*bc*_1_ construction
F_rellacZII	ATATTCTAGACTGGATGCGCGCGGTTTC	*rel*::*lacZ* translational fusion
R_rellacZII	ATATATCGATAGACTGCACGGCCTGACC	*rel*::*lacZ* translational fusion
F_eislacZII	ATATTCTAGACCAAGGCATGGTTGGGCAC	*eis*::*lacZ* translational fusion
R_eislacZII	ATATGGATCCCGGGCTACACAGGGTCACAGTC	*eis*::*lacZ* translational fusion
F_wag31lacZII	ATATTCTAGACCGTGACTGGCGTCCCAC	*wag31*::*lacZ* translational fusion
R_wag31lacZII	ATATATCGATGGCAGGTGTAAGCGGCATTGT	*wag31*::*lacZ* translational fusion
F_rel_His	ATTTCATATGGTCGACGAGCCAGGC	*rel* overexpression
R_rel_His	ATTTATCGATTCAGTGATGGTGATGGTGATGGGCCGCGCTGGTGACGCG	*rel* overexpression
F_sigA_RT	CTTGAGGTGACCGACGATCT	qRT-PCR
R_sigA_RT	AGCTTCTTCTTCCTCGTCCT	qRT-PCR
F_cydA_RT	CGGTGGCAGTTCGGAATCAC	qRT-PCR
R_cydA_RT	CAGAAAAAGTTTGCCGAAGAAACG	qRT-PCR
F_1441_RT	AAGGTGATCGCGAGCGCTG	qRT-PCR
R_1441_RT	GGAAGGCACGGCCCTGGG	qRT-PCR
F_1470_RT	TCGCAGAGCCCATCTCGGTC	qRT-PCR
R_1470_RT	GCGTGTACCCCTCGGTGACAC	qRT-PCR
F_1525_RT	GGGTCGTCCTCGCACCAG	qRT-PCR
R_1525_RT	GTTGTGCAGAGCACCCTTCTTGG	qRT-PCR
F_2654_RT	GCCGAGCAGAAAAAAGAGATCCTG	qRT-PCR
R_2654_RT	GCGACGTACTTCAGCAGGCG	qRT-PCR
F_6894_RT	CGTCGCCTCCCGTGGGG	qRT-PCR
R_6894_RT	CGTTGACCGACAGCGTGACAT	qRT-PCR

### Construction of plasmids

#### The temperature-sensitive suicide plasmids for the construction of mutant strains of *M. smegmatis*

To construct pKOTsΔrel, we performed two rounds of recombination PCR. Using the chromosomal DNA of *M. smegmatis* as a template, two primary PCR reactions were conducted. The first reaction utilized the primers F_relmut and R_relrec, while the second used the primers F_relrec and R_relmut, resulting in two 38-bp overlapping DNA fragments (401 and 384 bp, respectively). Both PCR products contain the same 540-bp deletion within *rel* in the overlapping region. In the secondary PCR, a 747-bp DNA fragment with a deletion in the *rel* gene was obtained using both the primary PCR products as templates, along with the primers F_relmut and R_relmut. The resulting secondary PCR product was restricted with NotI and HindIII and subsequently cloned into pKOTs, resulting in pKOTsΔrel.

To construct pKOTsΔmprA, a PCR reaction was carried out with the primers F_mprAmut and R_mprAmut using the chromosomal DNA of *M. smegmatis* as a template. The resulting 1,469-bp DNA fragment, encompassing the 406-bp upstream and 374-bp downstream regions of *mprA*, was digested with HindIII. Subsequently, the restricted PCR product was cloned into pUC19 that had been digested with SmaI and HindIII, resulting in pUC19mprA. A 135-bp DNA fragment within *mprA* was removed from pUC19mprA using XhoI. The linear plasmid was self-ligated through the XhoI recognition site, yielding pUC19ΔmprA. Using pUC19ΔmprA as a template, another PCR reaction was performed with the primers F_mprAmut and R_mprAmut, resulting in a 1,343-bp DNA fragment with a deletion in *mprA*. The obtained PCR product was then restricted with HindIII and cloned into pKOTs that had been digested with HindIII and EcoRV, resulting in pKOTsΔmprA.

To construct pKOTsΔbc_1_, we performed two rounds of recombination PCR. We used the chromosomal DNA of *M. smegmatis* as a template for two primary PCR reactions. The first reaction was performed with the primers F_ bc1mut and R_ bc1rec, while the second used the primers F_bc1rec and R_ bc1mut, resulting in two 36-bp overlapping DNA fragments (481 and 384 bp, respectively). Both PCR products contain the same 1,309-bp deletion within *qcrB* in the overlapping region. In the secondary PCR, an 829-bp DNA fragment with a deletion in *qcrB* was obtained using both the primary PCR products as templates, along with the primers F_bc1mut and R_bc1mut. The secondary PCR product was restricted with NotI and HindIII, and cloned into pKOTs, yielding pKOTsΔbc_1_.

#### pNCIIrel, pNCIIeis, and pNCIIwag31

The plasmid pNCIIrel is a *rel*::*lacZ* translational fusion plasmid that contains the 5′ portion (39 bp) of *rel* and the 397-bp DNA sequence upstream of *rel*. For the construction of pNCIIrel, a 456-bp DNA fragment was amplified using the chromosomal DNA of *M. smegmatis* as a template and the primers F_rellacZII and R_rellacZII. The PCR product was restricted with XbaI and ClaI and cloned into the promoterless *lacZ* vector pNCII, yielding pNCIIrel.

The plasmid pNCIIeis is an *eis*::*lacZ* translational fusion plasmid that contains the 5′ portion (24 bp) of *eis* and the 474-bp DNA sequence upstream of *eis*. To construct pNCIIeis, a 518-bp DNA fragment was amplified using the chromosomal DNA of *M. tuberculosis* as a template and the primers F_eislacZII and R_eislacZII. The PCR product was restricted with XbaI and BamHI and cloned into pNCII, yielding pNCIIeis.

The plasmid pNCIIwag31 is a *wag31*::*lacZ* translational fusion plasmid that contains the 5′ portion (18 bp) of *wag31* and the 463-bp DNA sequence upstream of *wag31*. To construct pNCIIwag31, a 501-bp DNA fragment was amplified using the chromosomal DNA of *M. tuberculosis* as a template and the primers F_wag31lacZII and R_wag31lacZII. The PCR product was restricted with XbaI and ClaI and cloned into pNCII, creating pNCIIwag31.

#### pMH201rel

The plasmid pMH201rel is a derivative of the pMH201 integration vector that contains the C-terminally His_6_-tagged *rel* gene. A 2,429-bp DNA fragment encompassing the *rel* gene and six His codons immediately before its stop codon was amplified by PCR with the primers F_rel_His and R_rel_His, using the chromosomal DNA of *M. smegmatis* as a template. The PCR product was restricted with NdeI and ClaI and cloned into pMH201 with an acetamide-inducible promoter, yielding pMH201rel.

### Construction of mutant strains of *M. smegmatis*

The Δ*rel*, Δ*aa*_3_Δ*rel*, Δ*mprA*, Δ*aa*_3_Δ*mprA*, and Δ*bc*_1_ deletion mutants of *M. smegmatis* were generated through allelic exchange mutagenesis, which utilized the corresponding pKOTs-derived suicide plasmids (pKOTsΔrel, pKOTsΔmprA, and pKOTsΔbc_1_). The mutagenesis was performed in the background of the WT or Δ*aa*_3_ mutant strain, following the procedure previously described (Jeong et al., 2013). In brief, the temperature-sensitive suicide plasmid was introduced into *M. smegmatis* by electroporation. Transformants were selected at 30°C (replication-permissive temperature) on 7H9-glucose agar plates containing hygromycin, and the selected transformants were grown in 7H9-glucose liquid medium supplemented with hygromycin for 3 days at 30°C. Heterogenotes of *M. smegmatis*, which were generated by a single recombination event, were selected for their hygromycin resistance on 7H9-glucose agar plates at 42°C (replication-non-permissive temperature). The selected heterogenotes were grown on 7H9-glucose medium without antibiotics for 3 days at 37°C. Isogenic homogenates were obtained from the heterogenotes after a second recombination by selecting them for sucrose resistance on 7H9-glucose agar plates containing 10% (w/v) sucrose at 37°C. The allelic exchange was verified by PCR using isolated genomic DNA.

### RNA sequencing analysis

Three biological replicate cultures of the Δ*aa*_3_, Δ*aa*_3_Δ*rel*, and Δ*aa*_3_Δ*mprΑ* strains were grown aerobically to OD_600_ of 0.45–0.5. Total RNA of each culture was isolated as described previously (Kim et al., 2010). rRNA was eliminated from the isolated total RNA using the NEBNext rRNA Depletion kit (Bacteria) (NEB, Ipswich, MA, United States). The RNA sequencing libraries were created using a TruSeq standard mRNA Sample Prep Kit (Illumina, San Diego, CA, United States). The libraries were quantified using a KAPA Library Quantification Kit (KAPA Biosystems, Wilmington, MA, United States) for Illumina Sequencing platforms according to the qPCR Quantification Protocol Guide and qualified using the TapeStation D1000 ScreenTape (Agilent, Santa Clara, CA, United States). Indexed libraries were subsequently submitted to an Illumina NovaSeq (Illumina), and the paired-end (2 × 101 bp) sequencing of the nine libraries was conducted on an Illumina NovaSeq 6,000 platform at Macrogen Inc. (Seoul, South Korea) using the NovaSeq 6,000 sequencing protocol (Illumina). Paired-end reads (101 bp) were then mapped to the reference genome sequence of *M. smegmatis* mc^2^155 (GCF_000015005.1_ASM1500v1) with the program Bowtie 1.1.2 using default settings. Based on principal component analysis of the gene expression patterns in the *M. smegmatis* strains, one result from each of the Δ*aa*_3_, Δ*aa*_3_Δ*rel*, and Δ*aa*_3_Δ*mprΑ* strains was considered as an outlier and therefore excluded from subsequent analyses. The RNA sequencing data for the Δ*aa*_3_, Δ*aa*_3_Δ*rel*, and Δ*aa*_3_Δ*mprΑ* strains of *M. smegmatis* were deposited in NCBI’s Gene Expression Omnibus and are accessible through the GEO Series accession number GSE267048.

The RNA sequencing data for the WT and Δ*aa*_3_ strains of *M. smegmatis* strains have been previously deposited in NCBI’s Gene Expression Omnibus with the GEO Series accession number GSE155251 (Oh et al., 2020).

### *β*-galactosidase assay and determination of the protein concentration

The *β*-galactosidase activity was measured spectrophotometrically as described previously (Oh and Kaplan, 1999). The protein concentration was determined using a Bio-Rad protein assay kit (Bio-Rad, Hercules, CA, United States) with bovine serum albumin as the standard protein.

### Quantitative real-time PCR

RNA isolation from *M. smegmatis* strains and cDNA synthesis were performed as described elsewhere (Kim et al., 2010), with the exception of using a random hexamer primer (Thermo Fisher Scientific, Waltham, MA, United States) in place of gene-specific primers in cDNA synthesis. The presence of DNA contamination in the isolated RNA was assessed through PCR using the primers intended for use in quantitative real-time PCR (qRT-PCR). To determine the transcript levels of *MSMEG_1441, MSMEG_1470, MSMEG_1525, MSMEG_2654, MSMEG_6894, cydA* and *sigA*, qRT-PCR was performed in a 20-μl mixture. The mixture contained 5 μL of the template cDNA, 1 μL (15 pmol) of each of two gene-specific primers, 10 μL of TB GreenTM Premix Ex TaqTM (Tli RNase Plus) (Takara, Tokyo, Japan), 0.4 μL of the ROX passive fluorescent dye, and 2.6 μL of distilled water. The thermal cycling process commenced with 1 cycle at 95°C for 2 min, followed by 40 cycles of 95°C for 5 s and 64°C for 30 s. The *sigA* gene, which encodes the principal sigma factor, was employed as a reference gene for qRT-PCR to normalize the expression levels of *MSMEG_1441, MSMEG_1470, MSMEG_1525, MSMEG_2654, MSMEG_6894*, and *cydA*. Melting curve analysis was conducted for each reaction to verify the amplification of a single PCR product during qRT-PCR. The primers used for qRT-PCR are listed in Table 2.

### Western blotting analysis

Cell-free crude extracts were subjected to SDS-PAGE, and proteins on the gel were transferred to polyvinylidene fluoride membranes (Millipore, Burlington, MA, United States). Western blotting was performed as described previously (Mouncey and Kaplan, 1998). For detection of His_6_-tagged Rel, a mouse monoclonal IgG against His_6_ (Thermo Fisher Scientific; MA1-21315) was used at a 1:2,000 dilution. A horseradish peroxidase (HRP)-conjugated anti-mouse IgG (Bio-Rad) was used at a 1:10,000 for the detection of the primary antibody. The ECL kit (Advansta, San Jose, CA, United States) was used to visualize protein bands via a ChemiDoc imaging system (Bio-Rad).

### Ribosome profiling using sucrose density gradient sedimentation

The WT and Δ*aa*_3_ strains of *M. smegmatis* were grown aerobically to an OD_600_ of 0.45 to 0.5 in 50 mL of 7H9-glucose medium. After harvesting the bacterial cells by centrifugation, the cell pellets were washed with 1 mL of BP buffer [20 mM Tris–HCl (pH 7.5), 10 mM MgCl_2_, 100 mM NH_4_Cl, and 5 mM *β*-mercaptoethanol] and then resuspended in 0.5 mL of BP buffer. Cells were broken three times using a Fastprep FP120 (Thermo Fisher Scientific), and cell-free lysates were obtained by centrifugation at 12,000 × *g*, for 15 min at 4°C. Then, 0.5 mL of cleared lysates from the WT and Δ*aa*_3_ strains, with their OD_260_ values adjusted to be identical, were added to 10 mL of a 5–40% sucrose gradient in BP buffer and resolved by ultracentrifugation at 4°C in a Beckman SW41 rotor for 2.5 h at 37,000 rpm. The sucrose density gradient solution was eluted from the bottom of the centrifuge tube, and the presence of polysomes, ribosomes, and ribosomal subunits was monitored at 254 nm. The amounts of polysomes, assembled 70S ribosomes, and ribosomal subunits were compared between the WT and Δ*aa*_3_ strains after normalizing the ribosome profiles to the absorbance of ultraviolet (UV: 254 nm)-absorbing fractions containing proteins, nucleic acids, nucleotides, and other components.

### Statistical analysis

Results were subjected to statistical analysis using GraphPad Prism 10.0 (GraphPad software Inc., La Jolla, CA, United States). At least three biological replicates were used for each experiment. The data were evaluated statistically using an unpaired Student *t*-test. Differences were considered statistically significant at *p* < 0.05.

## Results

### Induction of SR under conditions that inhibit the respiratory ETC

**Figure 1:**
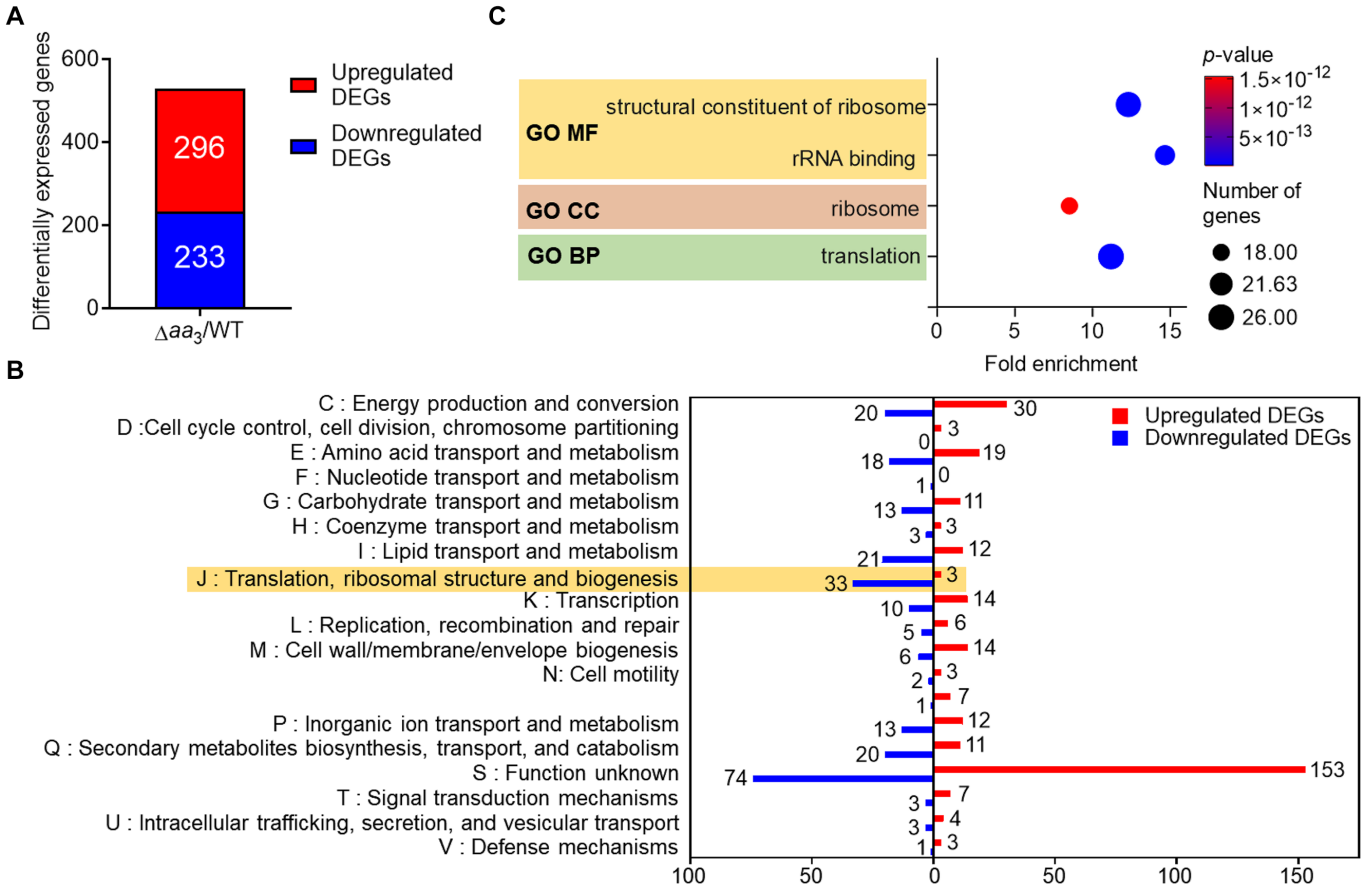
Comparative RNA sequencing analyses on the WT and Δ*aa*_3_ mutant strains of *M. smegmatis* grown aerobically to an OD_600_ of 0.45–0.5 in 7H9-glucose medium. **(A)** Identification of DEGs (│log_2_ FC in gene expression│ > 1, *p* < 0.05) in the Δ*aa*_3_ mutant relative to the WT strain. The number of the DEGs with increased (red) and decreased (blue) expression in the mutant, compared to the WT strain, is denoted. **(B)** COG analysis of the identified DEGs. The *y-*axis represents the COG category, and the *x-*axis indicates the number of the DEGs belonging to the corresponding category. The red and blue bars denote the DEGs with increased and decreased expression in the mutant, respectively, relative to the WT strain. **(C)** GO term enrichment analysis was performed on the DEGs with decreased expression in the mutant relative to the WT strain (log_2_FC in gene expression < −1, *p* < 0.05). The analysis was performed in terms of molecular function (MF), cellular component (CC), and biological process (BP). The size of each circle represents the number of enriched genes in each term, while the color of the circle denotes the *p*-value. A statistical criterion of *p* < 0.05 was used for each term to determine whether it is enriched for the input genes.

*M. tuberculosis* in granulomas encounters conditions inhibiting the respiration ETC, such as hypoxia, nutrient deprivation, nitrosative stress, and low pH, etc. (Timm et al., 2003; Baker and Abramovitch, 2018). These conditions have been considered signals for *M. tuberculosis* to transition into a dormant state. To gain insights into metabolic and physiological changes in mycobacteria exposed to conditions inhibiting the respiratory ETC, we performed comparative RNA sequencing analysis on the WT strain of *M. smegmatis* and its isogenic Δ*aa*_3_ mutant strain with a deletion in the *ctaC* gene encoding subunit II of the *aa*_3_ cytochrome *c* oxidase (Oh et al., 2020). The Δ*aa*_3_ mutant strain has an impaired growth rate and 50% respiration inhibition compared to the WT strain (Jeong et al., 2018). As shown in Figure 1A, we identified 529 differentially expressed genes (DEGs) whose expression was altered in the Δ*aa*_3_ mutant strain by more than |log_2_ fold change (FC)| > 1 with a *p*-value less than 0.05 relative to the WT strain (Supplementary Table S1). Among the identified DEGs, 296 were upregulated and 233 were downregulated in the Δ*aa*_3_ mutant relative to the WT strain. We carried out clusters of orthologous group (COG) analysis with the identified DEGs to gain insights into changes in the Δ*aa*_3_ mutant strain. The COG analysis was conducted based on eggNOG v5.0^1^ (Huerta-Cepas et al., 2019) (Figure 1B). Except for the “S” category representing function unknown, the “J” category exhibited the largest number of DEGs with the reduced expression in the Δ*aa*_3_ mutant relative to the WT strain. The “J” category encompasses genes related to translation, ribosomal structure and biogenesis. The second-largest number of downregulated DEGs belonged to the “I” category consisting of genes related to lipid transport and metabolism. Excluding the “S” category, the largest number of DEGs with the increased expression in the mutant relative to the WT strain belonged to the “C” category, which is associated with energy production and conversion.

To gain further insights into the functions associated with DEGs downregulated in the Δ*aa*_3_ mutant compared to the WT strain, enrichment of gene ontology (GO) in terms of molecular function (MF), cellular component (CC), and biological process (BP) was assessed using the DAVID functional annotation tool^2^ (Huang da et al., 2009; Sherman et al., 2022) (Figure 1C). Genes related to translation and ribosomes, such as ribosomal protein genes, were statistically significantly enriched in three GO terms. The decreased expression of genes related to ribosomes and translation is a representative phenomenon of SR (Prusa et al., 2018; Bange et al., 2021), suggesting the possibility of SR induction in *M. smegmatis* under conditions that inhibit the respiratory ETC.

**Figure 2:**
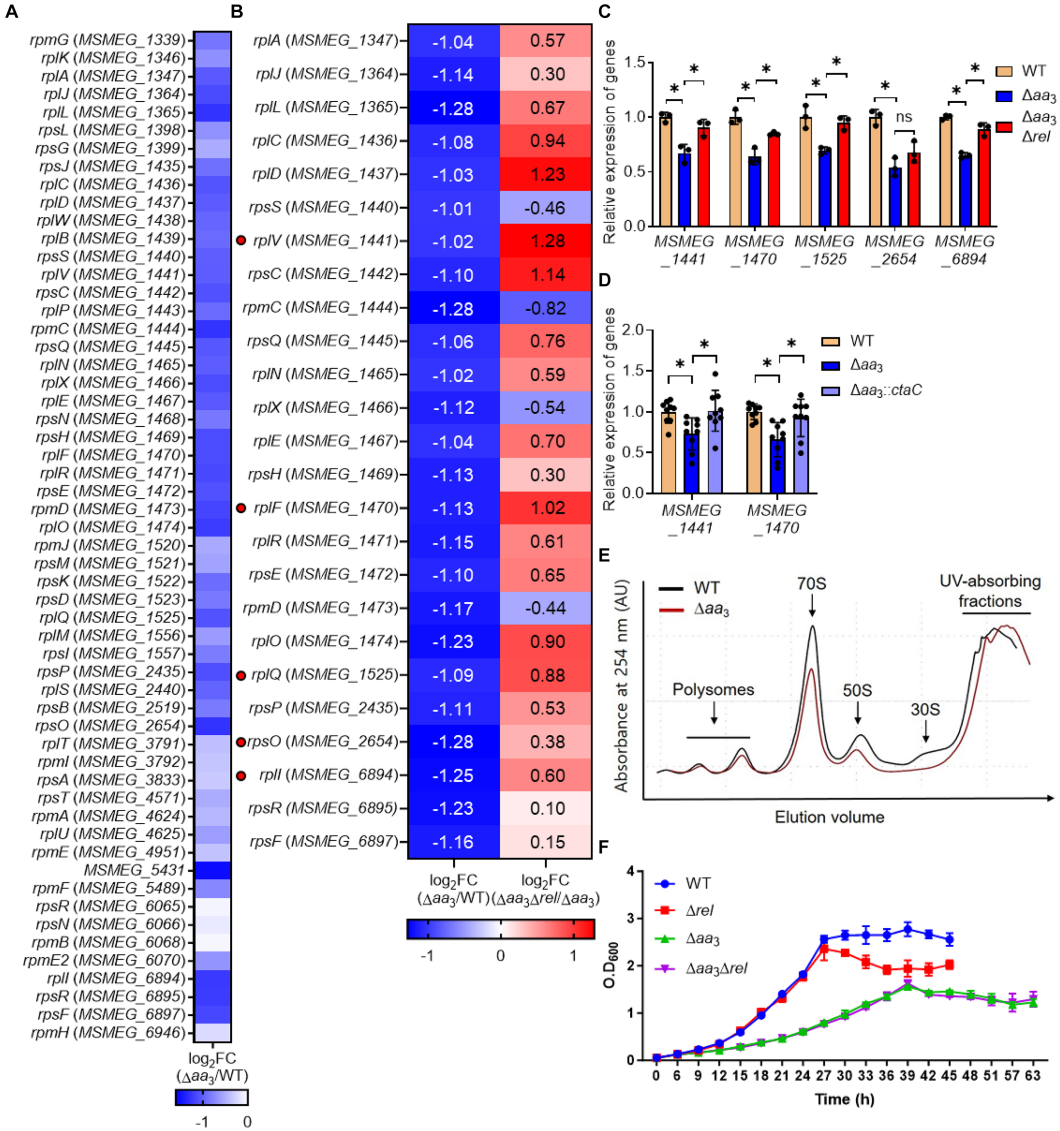
Rel-dependent decrease in the expression of the ribosomal protein genes in the Δ*aa*_3_ mutant strain relative to the WT strain and the growth curves of the WT, Δ*rel*, Δ*aa*_3_, and Δ*aa*_3_Δ*rel* strains. **(A)** The heatmap showing the relative expression of 56 ribosomal protein genes in the Δ*aa*_3_ mutant relative to the WT strain. The relative expression levels are expressed as log_2_ FC in gene expression for the ribosomal protein genes. **(B)** The heatmap showing the log_2_ FC in expression of 25 ribosomal protein genes that are significantly downregulated (log_2_ FC in gene expression < −1) in the Δ*aa*_3_ mutant relative to the WT strain [log_2_ FC (Δ*aa*_3_/WT)]. The heatmap also shows the log_2_ FC in expression of the 25 ribosomal protein genes in the Δ*aa*_3_Δ*rel* mutant relative to the Δ*aa*_3_ mutant [log_2_ FC (Δ*aa*_3_Δ*rel*/Δ*aa*_3_)]. The genes selected for validation by qRT-PCR are marked on the left of the heatmap with red circles. **(C)** Validation of the expression levels of the selected ribosomal protein genes (*MSMEG_1441*, *MSMEG_1470*, *MSMEG_1525*, *MSMEG_2654*, *MSMEG_6894*) in the WT, Δ*aa*_3_, and Δ*aa*_3_Δ*rel* strains. The expression levels of the genes were quantitatively determined by qRT-PCR and normalized to *sigA* (the gene encoding the principal sigma factor) expression. The expression level of each ribosomal protein gene in the WT strain is set at 1, and the relative values are expressed for the mutant strains. **(D)** Complementation of the Δ*aa*_3_ mutant with the *ctaC* gene in terms of the expression of *MSMEG_1441* and *MSMEG_1470*. For complementation of the Δ*aa*_3_ mutant, pMV306ctaC (a pMV306-derived plasmid carrying the intact *ctaC* gene and its own promoter) was introduced into the mutant. As control strains, the WT and mutant strains with the empty vector pMV306 were used in the experiment. The expression levels of the genes were quantitatively determined by qRT-PCR and normalized to the expression of *sigA*. The expression level of *MSMEG_1441* or *MSMEG_1470* in the WT strain with pMV306 is set at 1, and the relative values are expressed for the mutant and complemented strains. **(E)** Ribosome profiling of the WT and Δ*aa*_3_ strains. The amounts of polysomes, assembled 70S ribosomes, and ribosomal subunits were compared between the WT and Δ*aa*_3_ strains after normalizing the ribosome profiles to the absorbance of UV (254 nm)-absorbing fractions containing proteins, nucleic acids, nucleotides, and other components. **(F)** Growth curves of the WT, Δ*rel*, Δ*aa*_3_, and Δ*aa*_3_Δ*rel* strains in 7H9-glucose medium under aerobic conditions. All values are the means of the results from three biological replicates for panels **(A,C,F)** and nine biological replicates for panel **(D)**. The comparative RNA sequencing results between the Δ*aa*_3_ and Δ*aa*_3_Δ*rel* strains shown in panel **(B)** were obtained from two biological replicates as described in section Materials and Methods. The error bars indicate the standard deviations. *p < 0.05. AU, arbitrary unit.

We compared the relative expression levels of a total of 56 genes encoding the ribosomal proteins using the RNA sequencing data from the WT vs. Δ*aa*_3_ strains. As shown in Figure 2A, the expression of most ribosomal protein genes was downregulated in the Δ*aa*_3_ mutant strain relative to the WT strain. To examine whether the decreased expression of the ribosomal protein genes in the Δ*aa*_3_ mutant is due to the Rel-dependent induction of SR, we compared the expression levels of 25 selected ribosomal protein genes, whose expression was decreased by less than −1 of log_2_ FC in the Δ*aa*_3_ mutant relative to the WT strain, between the Δ*aa*_3_ and Δ*aa*_3_Δ*rel* mutant strains. In the Δ*aa*_3_Δ*rel* mutant, the *rel* gene encoding the major (p)ppGpp synthetase/hydrolase was null-mutated in the background of the Δ*aa*_3_ mutant. As shown in Figure 2B, the decreased expression of the ribosomal protein genes observed in the Δ*aa*_3_ mutant strain was at least partly restored in the Δ*aa*_3_Δ*rel* mutant strain. We selected five genes that do not form an operon with each other and performed qRT-PCR to confirm the RNA sequencing results. The qRT-PCR results were consistent with those of the comparative RNA sequencing (Figure 2C). In order to confirm that the decreased expression of ribosomal protein genes in the Δ*aa*_3_ mutant strain is due to the deletion of *ctaC,* the Δ*aa*_3_ mutant strain was complemented with the intact *ctaC* gene using the *ctaC*-expressing plasmid pMV306ctaC. As shown in Figure 2D, the introduction of pMV306ctaC into the Δ*aa*_3_ mutant led to the restoration of the expression of *MSMEG_1441* and *MSMEG_1470* to the WT level. This indicates that the decreased expression of the ribosomal protein genes in the Δ*aa*_3_ mutant relative to the WT strain is attributed to the inactivation of the *aa*_3_ oxidase. Ribosome profiling analysis showed that the amounts of polysomes, assembled 70S ribosomes, and ribosomal subunits were decreased in the Δ*aa*_3_ mutant, compared to the WT strain (Figure 2E). Taken together, RNA sequencing, qRT-PCR, and ribosome profiling results suggest that the expression of ribosomal protein genes and the biogenesis of ribosomes are decreased in the Δ*aa*_3_ mutant compared to the WT strain, and that this decrease in the Δ*aa*_3_ mutant strain occurs, at least in part, in a Rel-dependent way.

As judged by the growth curves of the WT and Δ*rel* strains, the inactivation of Rel in the WT strain did not affect its aerobic growth in 7H9-glucose medium until the stationary phase (Figure 2F). However, the optical density of the Δ*rel* mutant declined during the early stationary phase, while that of the WT strain was maintained, indicating that the absence of Rel and the resulting lack of SR induction are likely detrimental to the WT strain of *M. smegmatis* during the stationary phase. This observation is consistent with the previous report (Dahl et al., 2005). In contrast, the growth of the Δ*aa*_3_Δ*rel* strain was not different from that of Δ*aa*_3_ strain throughout all growth phases, indicating that the presence of Rel is neither beneficial nor detrimental to the growth of *M. smegmatis* under conditions that inhibit the respiratory ETC (Figure 2F).

**Figure 3:**
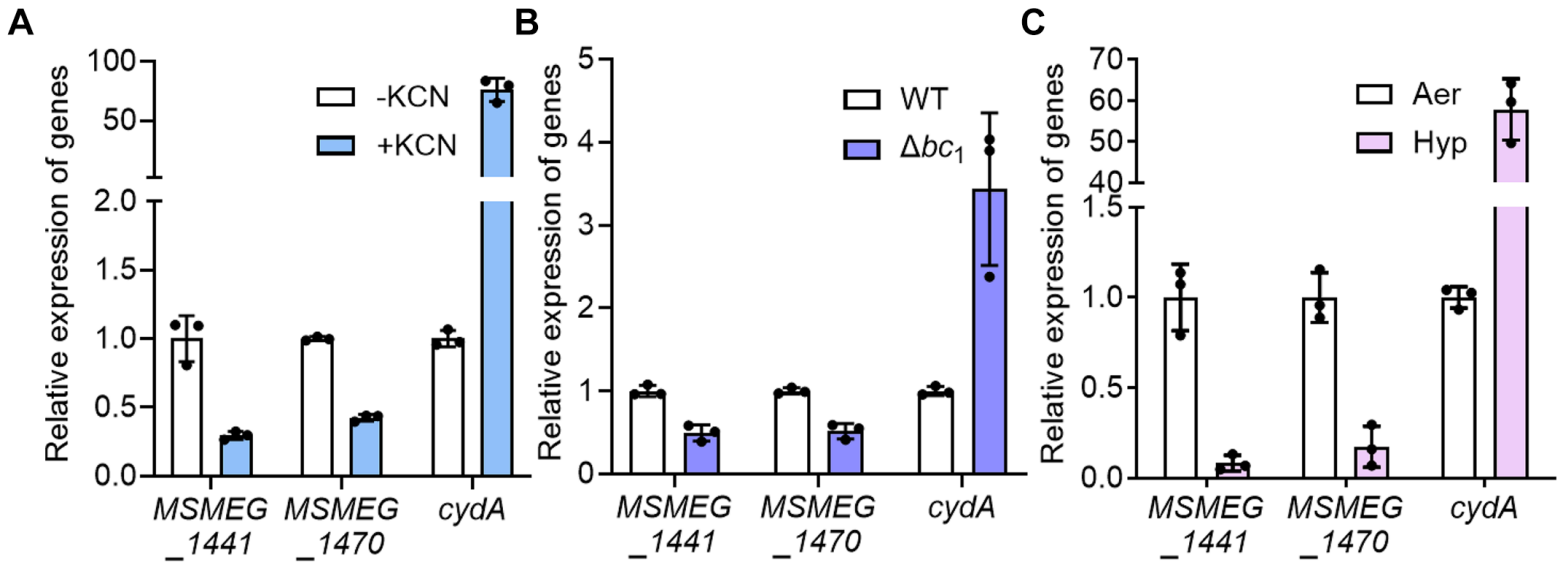
Effects of inhibiting the respiratory ETC on the expression of *MSMEG_1441* and *MSMEG_1470*. **(A)** The expression levels of *cydA* and the two ribosomal protein genes in the WT strain with or without KCN treatment. The WT strains were aerobically grown to an OD_600_ of 0.45–0.5 and further grown for 2 h either after treatment with 50 μM KCN (+KCN) or without KCN treatment as a control (−KCN). **(B)** The expression levels of *cydA* and the two ribosomal protein genes in the WT and Δ*bc*_1_ mutant strains grown aerobically to an OD_600_ of 0.45–0.5. **(C)** The expression levels of *cydA* and the two ribosomal protein genes in the WT grown under aerobic (Aer) or hypoxic (Hyp) conditions. The expression levels of *cydA*, *MSMEG_1441*, and *MSMEG_1470* were determined by qRT-PCR and normalized to that of *sigA*. The expression level of each gene in the control WT strains is set at 1, and the relative values are expressed for the KCN-treated, hypoxically grown WT, and the Δ*bc*_1_ mutant. All values provided are the averages of the results from three biological replicates. The error bars indicate the standard deviations.

**Figure 4:**
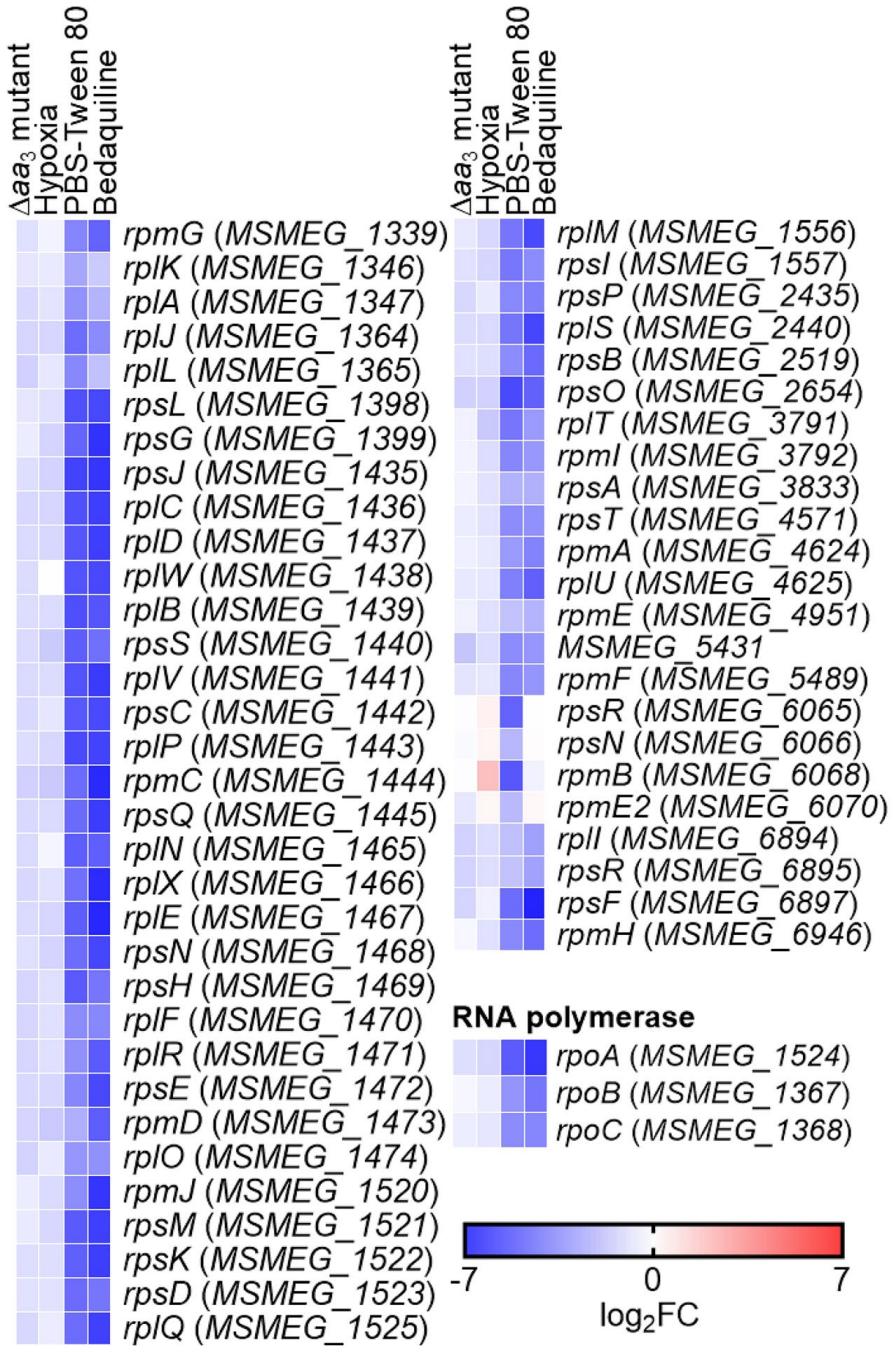
Transcriptional profiles of the genes encoding the ribosomal protein and RNA polymerase in *M. smegmatis* under respiration-inhibitory conditions. The heatmap shows the relative expression of the ribosomal protein and RNA polymerase genes in the WT strain of *M. smegmatis* exposed to various stress conditions [treatment of bedaquiline, hypoxia, and starvation (PBS-Tween 80)] that are expected to inhibit the respiratory ETC, compared to the WT strain grown aerobically without exposure to the stress conditions (control). The expression of the ribosomal protein and RNA polymerase genes in the Δ*aa*_3_ mutant of *M. smegmatis* relative to the WT strain is also included in the heatmap. The transcriptomic data used in the generation of the heatmap were retrieved from NCBI’s Gene Expression Omnibus using the following accession number: Δ*aa*_3_ mutant (GSE155251), hypoxia (GSE128412), PBS-Tween 80 (GSE69983), bedaquiline (GSE59871). The color and shading of each cell in the heatmap denote the log_2_ FC in gene expression in the experimental groups vs. the control group. The WT and Δ*aa*_3_ mutant strains of *M. smegmatis* were grown to an OD_600_ of 0.45–0.5 in 7H9-glucose medium (Oh et al., 2020). The transcriptomic data were obtained from the WT strain of *M. smegmatis* exposed to 2 mg/L of bedaquiline for 60 min (Hards et al., 2015), hypoxic conditions for 24 h (Martini et al., 2019), or PBS-Tween 80 medium for 60 min (Wu et al., 2016).

To confirm that the expression of ribosomal protein genes is reduced when the respiratory ETC is inhibited, we examined the expression of two selected ribosomal protein genes (*MSMEG_1441* and *MSMEG_1470*) in the WT strain treated with KCN, which inhibits the *aa*_3_ cytochrome *c* oxidase, as well as in the Δ*bc*_1_ mutant strain, where the *bcc*_1_ complex of the respiratory ETC is inactivated by deletion of the *qcrB* gene. The cytochrome *bcc*_1_ complex comprises the *bcc*_1_-*aa*_3_ branch of the ETC with the *aa*_3_ cytochrome *c* oxidase (Megehee et al., 2006). As a positive control gene that is induced under respiration-inhibitory conditions, we included the *cydA* gene, which encodes the catalytic subunit of the *bd* quinol oxidase, in the experiment. As shown in Figure 3A, the expression of *cydA* was significantly increased under the KCN-treated WT strain relative to the untreated control WT strain. In contrast, the expression of the ribosomal protein genes was reduced in the KCN-treated WT strain compared to the untreated WT strain. Similarly, the expression levels of the ribosomal protein genes in the Δ*bc*_1_ mutant strain was decreased to approximately 50% of those in the WT strain, while the expression of *cydA* was significantly increased in the Δ*bc*_1_ mutant strain relative to the WT strain (Figure 3B). We further examined whether the expression of the ribosomal protein genes is decreased in the WT strain when exposed to hypoxic conditions that inhibit the respiratory ETC. As depicted in Figure 3C, the expression levels of the ribosomal protein genes were decreased by more than 80% under hypoxic conditions relative to aerobic conditions, whereas the expression level of *cydA* was greatly increased under hypoxic conditions compared to aerobic conditions. By compiling several deposited transcriptomic datasets obtained from *M. smegmatis* exposed to conditions likely to inhibit the respiratory ETC such as hypoxia, bedaquiline (an inhibitor of ATP synthase) treatment, and starvation (PBS-Tween 80) due to a lack of the final electron acceptor O_2_, the coupling of the ETC and ATP synthase, and a lack of electron donors, respectively, we observed that the expression of the genes encoding the ribosomal proteins and RNA polymerase was decreased under the tested conditions, similar to the Δ*aa*_3_ mutant (Figure 4). The decrease effect was more noticeable under the well-known SR-inducing starvation condition relative to the Δ*aa*_3_ mutant, indicating that the extent of SR induction in the Δ*aa*_3_ mutant is not as strong as that under starvation conditions. Collectively, these results confirm a decrease in the expression of ribosomal protein genes under conditions that inhibit the respiratory ETC.

**Figure 5:**
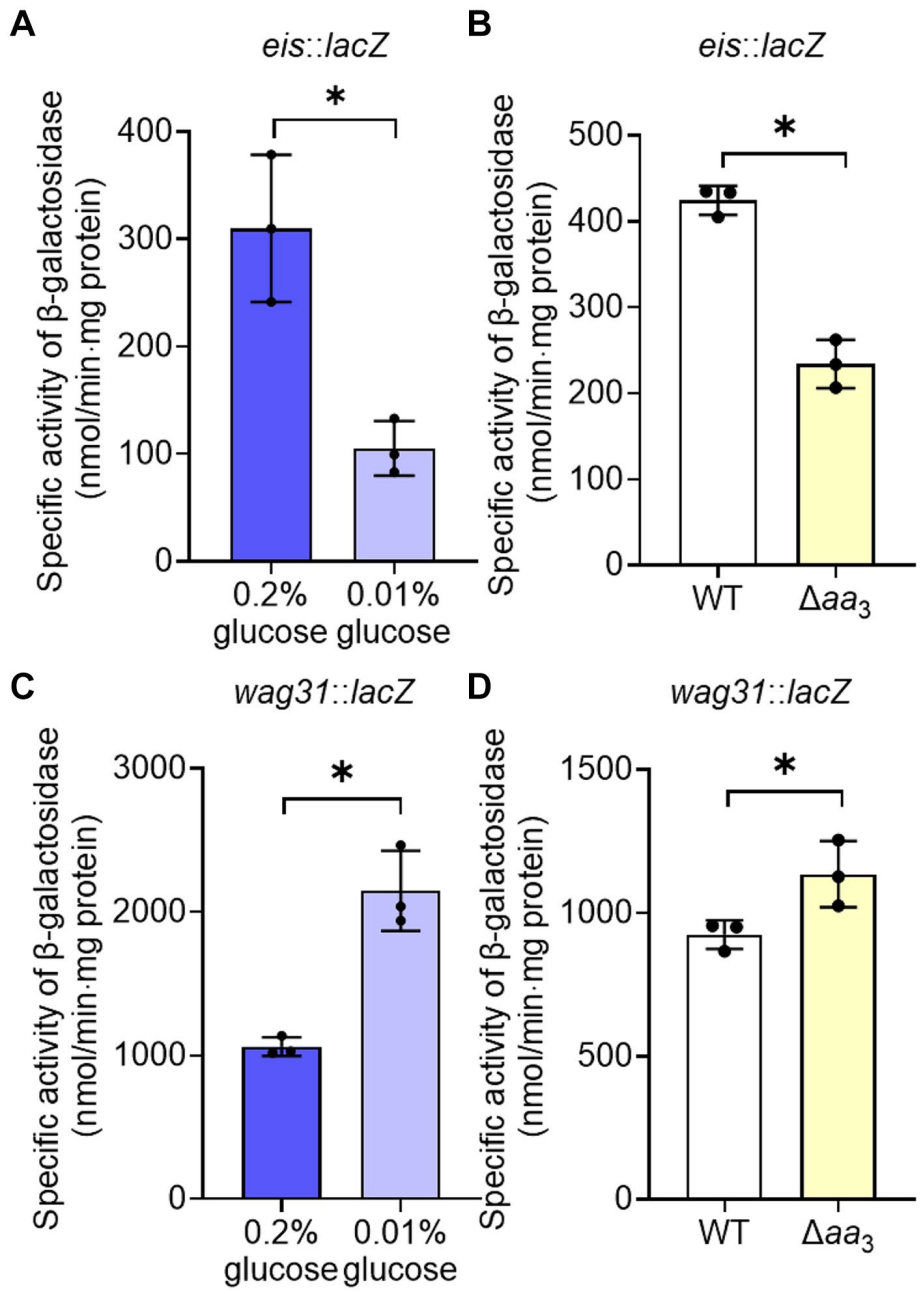
Verification of SR induction in the Δ*aa*_3_ mutant of *M. smegmatis* by determining the expression levels of the *eis* and *wag31* genes. **(A)** Expression of *eis* in the WT strain grown under glucose-replete (0.2%) and glucose-limiting (0.01%) conditions. **(B)** Expression of *eis* in the WT and Δ*aa*_3_ mutant strains. **(C)** Expression of *wag31* in the WT strain grown under glucose-replete and glucose-limiting conditions. **(D)** Expression of *wag31* in the WT and Δ*aa*_3_ mutant strains. The WT and Δ*aa*_3_ mutant strains containing the *eis*::*lacZ* and *wag31*::*lacZ* translational fusion plasmids, pNCIIeis and pNCIIwag31, were used to measure the expression levels of *eis* and *wag31*, respectively. The WT and Δ*aa*_3_ mutant strains were grown aerobically to an OD_600_ of 0.45 to 0.5 in 7H9-glucose medium. Cell-free crude extracts were used to measure *β*-galactosidase activity. All values provided were determined from three biological replicates. The error bars indicate the standard deviations. *p < 0.05.

The observed decrease in the expression of the ribosomal protein genes in the Δ*aa*_3_ mutant strain in a Rel-dependent manner led us to assume that SR is induced under respiration-inhibitory conditions. Because it was difficult for us to quantitatively measure changes in intracellular (p)ppGpp levels in the Δ*aa*_3_ mutant where a strong SR does not occur, we instead examined whether SR is induced in the Δ*aa*_3_ mutant strain by comparatively determining the expression of the *eis* (*Rv2416c*) and *wag31* (*Rv2145c*) genes of *M. tuberculosis* in the WT and Δ*aa*_3_ mutant strains of *M. smegmatis*. The *eis* gene encodes the enhanced intracellular survival protein that was suggested to be involved in suppressing host innate immune defenses by modulating autophagy, inflammatory responses, and cell death (Dahl et al., 2005; Shin et al., 2010). The *wag31* gene encodes the DivIVA protein that regulates cell shape and cell division in Gram-positive bacteria (Cha and Stewart, 1997; Flardh, 2003). The expression of the *eis* and *wag31* genes of *M. tuberculosis* has been previously demonstrated to be decreased and increased, respectively, in *M. smegmatis* under SR-inducing conditions in a Rel-dependent manner, when the genes with their own promoters were introduced into *M. smegmatis* (Dahl et al., 2005; Dahl and Lau Bonilla, 2011). First, we reaffirmed whether the expression of the *eis* and *wag31* genes is altered in *M. smegmatis* under a nutrient starvation condition that induces the SR. The WT strains of *M. smegmatis* with the *eis*::*lacZ* or *wag31*::*lacZ* translational fusion plasmid (pNCIIeis or pNCIIwag31) were assessed for the expression levels of the two reporter genes after growth in 7H9 medium supplemented with 0.01% glucose for nutrient starvation and 0.2% glucose for the control (Figures 5A,C). As reported previously (Dahl et al., 2005; Dahl and Lau Bonilla, 2011), the expression level of *eis* was decreased, while that of *wag31* was increased under glucose starvation (0.01%) relative to those in the WT strain supplemented with 0.2% glucose. As shown in Figure 5B, the expression level of *eis* was decreased in the Δ*aa*_3_ mutant strain relative to that in the WT strain. In contrast, the expression level of *wag31* was slightly increased in the Δ*aa*_3_ mutant strain relative to that in the WT strain (Figure 5D). These results support our assumption that SR is to some extent induced in the Δ*aa*_3_ mutant.

### Increased expression of *rel* and induction of SR through the MprBA-SigE-SigB regulatory pathway under conditions that inhibit the respiratory ETC

**Figure 6:**
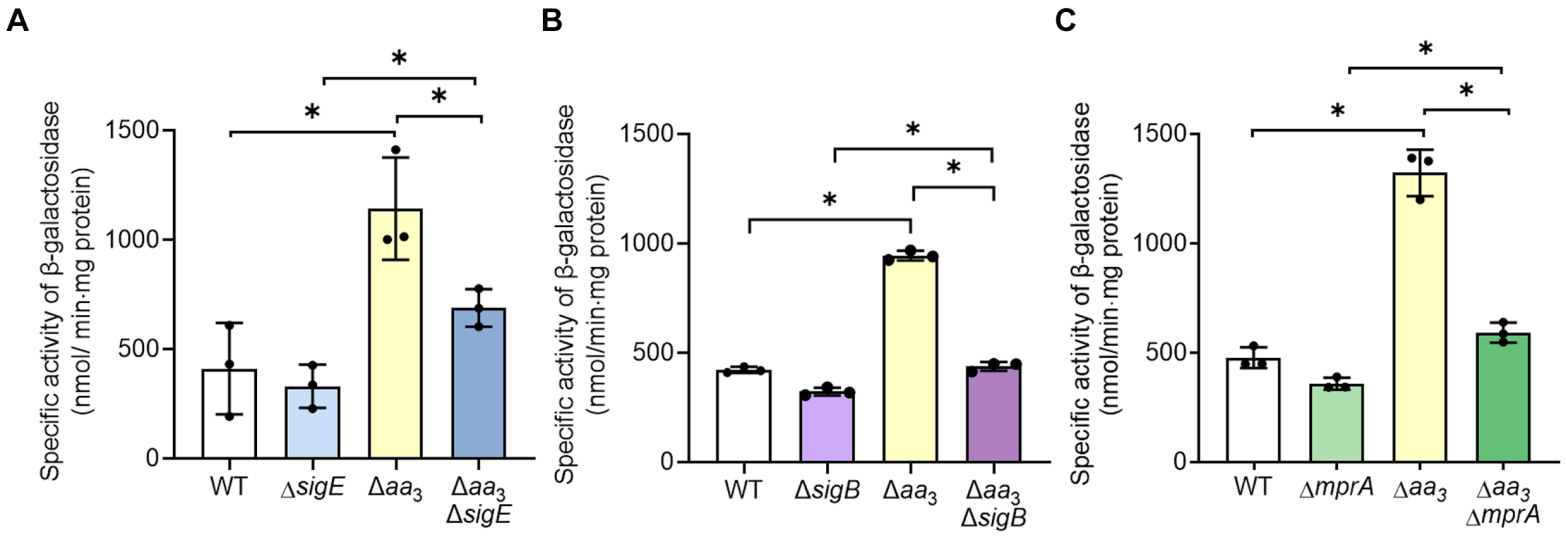
Involvement of the MprBA-SigE-SigB regulatory pathway in the upregulation of the *rel* gene in the Δ*aa*_3_ mutant of *M. smegmatis*. **(A)** Expression of *rel* in the WT, Δ*sigE*, Δ*aa*_3_, and Δ*aa*_3_Δ*sigE* strains. **(B)** Expression of *rel* in the WT, Δ*sigB*, Δ*aa*_3_, and Δ*aa*_3_Δ*sigB* strains. **(C)** Expression of *rel* in the WT, Δ*mprA*, Δ*aa*_3_, and Δ*aa*_3_Δ*mprA* strains. The expression level of the *rel* gene in the WT and mutant strains was measured using the *rel*::*lacZ* translational fusion plasmid pNCIIrel. The WT and mutant strains containing pNCIIrel were grown aerobically to an OD_600_ of 0.45 to 0.5 in 7H9-glucose medium. Cell-free crude extracts were used to measure *β*-galactosidase activity. All values provided were determined from three biological replicates. The error bars indicate the standard deviations. *p < 0.05.

Given the finding that SR is induced in the Δ*aa*_3_ mutant, we sought to investigate how SR is induced in the Δ*aa*_3_ mutant. One possibility is that the expression of a gene encoding the enzyme that produces (p)ppGpp is increased in the Δ*aa*_3_ mutant. There are two (p)ppGpp synthetases, Rel and RelZ, in *M. smegmatis* (Murdeshwar and Chatterji, 2012). According to our RNA sequencing results, the reads per kilobase per million mapped reads (RPKM) value of *rel* was 5.1-fold higher than that of *relZ* in the WT strain of *M. smegmatis* grown aerobically, indicating that Rel is the predominantly expressed (p)ppGpp synthetase in *M. smegmatis* (data not shown). Furthermore, our RNA sequencing results showed that the expression of *rel* was increased 1.3-fold in the Δ*aa*_3_ mutant relative to the WT strain, while the expression of *relZ* was not different between the WT and Δ*aa*_3_ mutant strains (data not shown). To verify whether the expression of *rel* is increased in the Δ*aa*_3_ mutant compared to the WT strain, we assessed the expression level of *rel* in the WT and Δ*aa*_3_ mutant strains using the *rel*::*lacZ* translational fusion pNCIIrel. As shown in Figure 6, the *β*-galactosidase assay showed that the expression of *rel* was more than two-fold higher in the Δ*aa*_3_ mutant relative to the WT strain when both strains were grown aerobically. The expression of *rel* is known to be regulated by the alternative sigma factor SigE. SigE was suggested to be one of the central nodes of the regulatory network involved in SR in mycobacteria (Sureka et al., 2007). Furthermore, it was demonstrated that transcription of *sigB*, which encodes another alternative sigma factor, strictly depends on SigE, and that the SigE-SigB regulatory pathway is activated in the Δ*aa*_3_ mutant of *M. smegmatis* (Oh et al., 2022). To examine whether the increased expression of *rel* in the Δ*aa*_3_ mutant is attributed to SigE and SigB, the expression of the *rel* gene was comparatively determined in the WT, Δ*sigE*, Δ*sigB*, Δ*aa*_3_, Δ*aa*_3_Δ*sigE*, and Δ*aa*_3_Δ*sigB* strains of *M. smegmatis* carrying pNCIIrel (Figures 6A,B). In the Δ*aa*_3_Δ*sigE* mutant, the expression level of *rel* was reduced to an intermediate level between those in the WT and Δ*aa*_3_ mutant strains (Figure 6A). The expression level of *rel* in the Δ*aa*_3_Δ*sigB* mutant was shown to be only slightly increased compared to that observed for the Δ*sigB* mutant (Figure 6B). Considering the hierarchical order of SigE and SigB in the SigE-SigB signaling pathway and the stricter dependence on SigB than SigE for the upregulation of *rel* expression in the Δ*aa*_3_ mutant, these results suggest that SigB, rather than SigE, is directly involved in the upregulation of *rel* in the Δ*aa*_3_ mutant. Since the MprA response regulator of the MprBA TCS is known to positively regulate the expression of *sigE* in mycobacteria (He et al., 2006), we assessed whether MprA is involved in the upregulation of *rel* expression in the Δ*aa*_3_ mutant. For this purpose, the expression level of *rel* was comparatively determined in the WT, Δ*mprA*, Δ*aa*_3_, and Δ*aa*_3_Δ*mprA* strains of *M. smegmatis* carrying pNCIIrel (Figure 6C). In the Δ*aa*_3_Δ*mprA* mutant, the increased expression of *rel* observed for the Δ*aa*_3_ mutant relative to the WT strain was almost abolished, which indicates that the MprBA TCS is required for the upregulation of *rel* expression caused by the inactivation of the *aa*_3_ oxidase. Taken together, the results presented in Figure 6 suggest that respiration inhibition such as the inactivation of the *aa*_3_ oxidase brings about an increase in *rel* expression through the MprBA-SigE-SigB signaling pathway.

**Figure 7:**
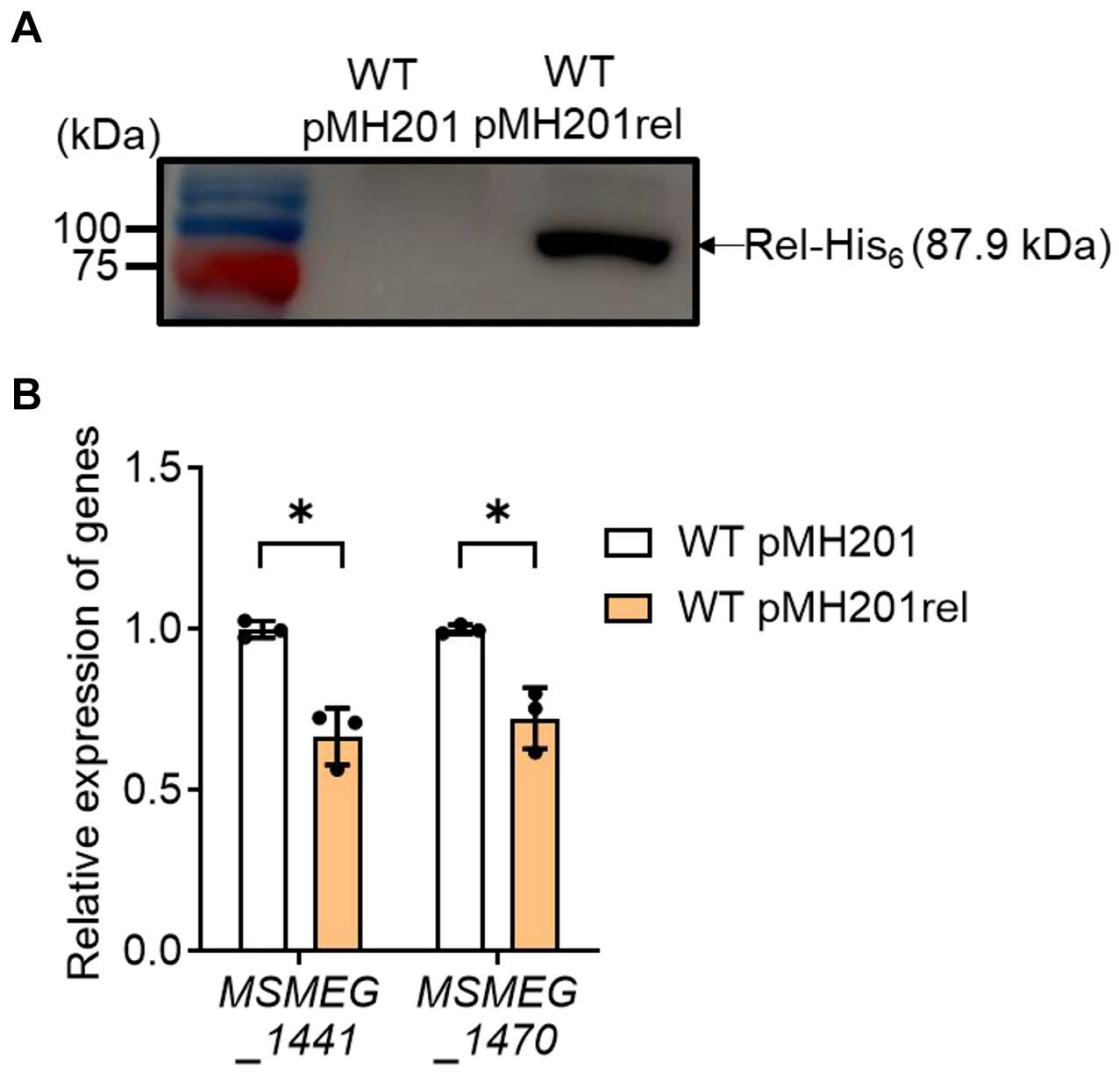
Effects of *rel* overexpression on the expression of *MSMEG_1441* and *MSMEG_1470* in the WT strain of *M. smegmatis*. The *rel* gene was overexpressed in the WT strain of *M. smegmatis* using pMH201rel that carries the C-terminally His_6_-tagged *rel* gene under the control of an acetamide-inducible promoter. As a control strain, the WT strain with the empty vector pMH201 was used in the experiment. **(A)** Crude extracts (10 μg) of the WT strains with pMH201 or pMH201rel were subjected to Western blotting analyses using a His-tag antibody to detect the C-terminally His_6_-tagged Rel. **(B)** The expression levels of *MSMEG_1441* and *MSMEG_1470* were quantitatively determined by qRT-PCR and normalized to that of *sigA*. The WT strains containing pMH201 or pMH201rel were grown aerobically to an OD_600_ of 0.45 to 0.5 in 7H9-glucose medium supplemented 0.1% (w/v) acetamide. The expression level of *MSMEG_1441* or *MSMEG_1470* in the WT strain with pMH201 is set at 1, and the relative values are expressed for the WT strain with pMH201rel. All values provided were determined from three biological replicates. The error bars indicate the standard deviations. *p < 0.05.

Next, we examined whether an increase in *rel* expression results in a decrease in the expression of ribosomal protein genes in the WT strain of *M. smegmatis* (Figure 7). The *rel* gene was overexpressed in the presence of acetamide using pMH201rel, in which the C-terminally His_6_-tagged *rel* gene is expressed by an acetamide-inducible promoter. Western blotting analysis using a His-tag antibody showed that the His_6_-tagged Rel protein was expressed in the WT strain carrying pMH201rel, while no band was observed at the corresponding position in case of the WT strain with the empty vector pMH201 (Figure 7A). The expression levels of *MSMEG_1441* and *MSMEG_1470* were reduced in the WT strain with pMH201rel by about 30% compared to those in the WT strain with pMH201 (Figure 7B), suggesting the possibility that the induction of SR in the Δ*aa*_3_ mutant is at least partly attributable to the increased expression of *rel* in the mutant.

**Figure 8:**
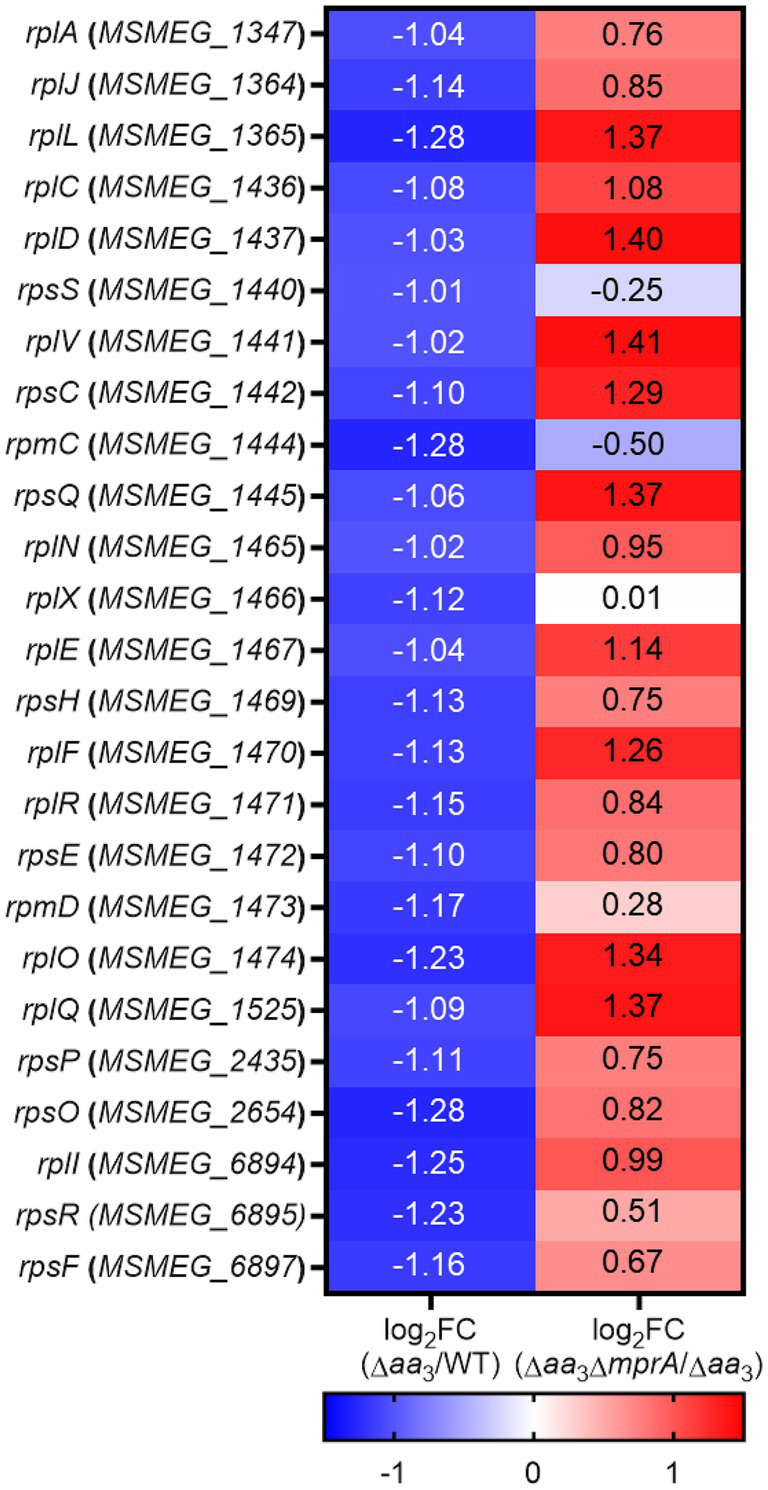
RNA sequencing results showing the involvement of the MprBA TCS in the downregulation of the ribosomal protein genes in the Δ*aa*_3_ mutant of *M. smegmatis*. The heatmap shows the log_2_ FC in expression of 25 ribosomal protein genes that are significantly downregulated (log_2_ FC in gene expression < −1) in the Δ*aa*_3_ mutant relative to the WT strain [log_2_ FC (Δ*aa*_3_/WT)], as well as the log_2_ FC in expression of the 25 ribosomal protein genes in the Δ*aa*_3_Δ*mprA* mutant relative to the Δ*aa*_3_ mutant [log_2_ FC (Δ*aa*_3_Δ*mprA*/Δ*aa*_3_)].

Given the positive correlation of the extent of *rel* expression with SR induction, as well as the abolishment of an increase in *rel* expression in the Δ*aa*_3_ mutant by the null mutation of *mprA*, we assumed that the SR induced in the Δ*aa*_3_ mutant could be abolished in the Δ*aa*_3_Δ*mprA* mutant. To examine this assumption, we compared the expression levels of 25 selected ribosomal protein genes, whose expression was decreased by less than −1 of log_2_ FC in the Δ*aa*_3_ mutant relative to the WT strain, between the Δ*aa*_3_ and Δ*aa*_3_Δ*mprA* mutant strains. As shown in Figure 8, the decreased expression of the ribosomal protein genes observed in the Δ*aa*_3_ mutant strain was mostly restored in the Δ*aa*_3_Δ*mprA* mutant strain.

## Discussion

When the SR is induced, it is known to affect many aspects of cellular metabolism, including central dogma processes such as transcription and translation. These alterations elicit physiological changes, such as a general slowdown of protein synthesis and growth (Legault et al., 1972; Corrigan et al., 2016), which aids bacterial adaptation and survival under stress conditions. The regulation of gene expression by SR in mycobacteria exhibits similarities to the conventional SR: the expression of genes related with ribosome biogenesis, as well as those encoding RNA polymerase, is downregulated in a Rel-dependent way under SR-inducing conditions (Stallings et al., 2009). In mycobacteria, SR has been demonstrated to be induced when they were exposed to nutrient starvation, oxidative stress, and hypoxic conditions (Avarbock et al., 2000; Primm et al., 2000; Betts et al., 2002; Stallings et al., 2009).

In this study, we presented several lines of evidence that support the induction of SR in *M. smegmatis* under conditions that inhibit the respiratory ETC. Our RNA sequencing results showed that the expression of most ribosomal protein genes and RNA polymerase genes was decreased in the aerobically grown Δ*aa*_3_ mutant strain compared to the WT strain grown under the same conditions. As in the Δ*aa*_3_ mutant, the expression levels of two representative ribosomal protein genes, *MSMEG_1441* and *MSMEG_1470*, were shown to decrease under conditions that inhibit the respiratory ETC, such as treatment of KCN, inactivation of the cytochrome *bcc*_1_ complex, and hypoxic conditions. Furthermore, the previous transcriptomic datasets have shown that the expression of the ribosomal protein genes is reduced in *M. smegmatis* treated with bedaquiline, as well as in *M. smegmatis* exposed to hypoxic conditions. These results support our assumption that inhibition of the respiratory ETC leads to SR induction in *M. smegmatis*. Furthermore, changes in the expression of the known SR-responsive genes *eis* and *wag31* in the Δ*aa*_3_ mutant corroborated our assumption that SR is indeed induced in the Δ*aa*_3_ mutant. The reduced expression of ribosomal protein genes in the Δ*aa*_3_ mutant was recovered by a null mutation of *rel*, which indicates Rel-dependent induction of SR in the Δ*aa*_3_ mutant. Our findings that the expression of *rel* is increased in the Δ*aa*_3_ mutant and that overexpression of *rel* leads to the decreased expression of two ribosomal protein genes, *MSMEG_1441* and *MSMEG_1470*, suggest that the elevated expression of *rel* under respiration-inhibitory conditions contributes to SR induction in *M. smegmatis*.

**Figure 9:**
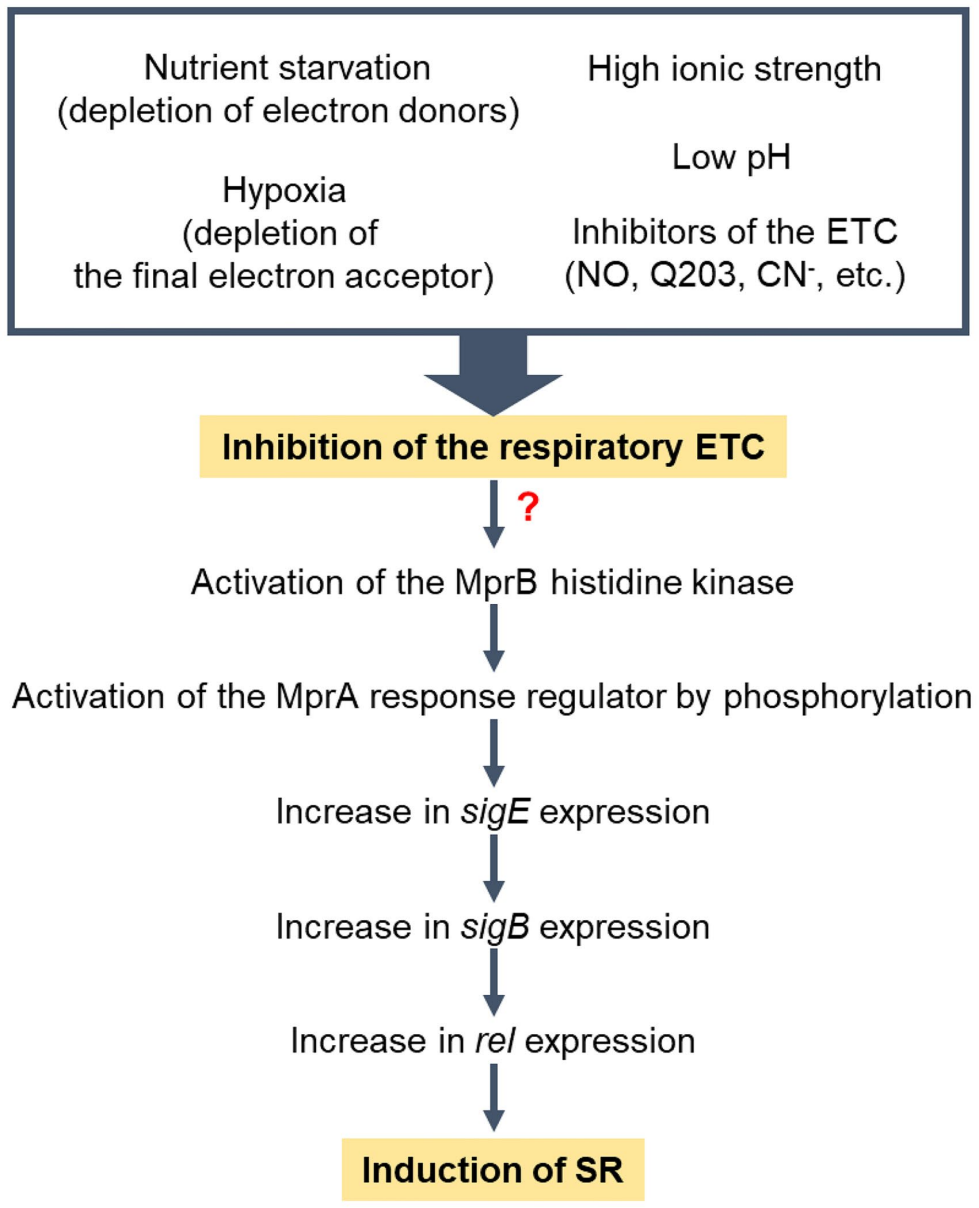
Model for the induction of SR in *M. smegmatis* under respiration-inhibitory conditions. “?” indicates that the mechanism by which the MprBA TCS is activated in response to inhibition of the respiratory ETC remains elusive. Low pH and high ionic strength have been demonstrated to inhibit the respiratory ETC of *M. smegmatis* (Oh and Oh, 2024).

Previously it has been reported that the expression of *rel* in *M. smegmatis* is regulated via the MprBA-SigE regulatory pathway, in which the MprBA TCS positively regulates the expression of *sigE* (He et al., 2006; Sureka et al., 2007). SigE was suggested to be an alternative sigma factor that participates in transcription of *rel* (Sureka et al., 2007). Our recent RNA sequencing analysis revealed that the expression of the SigE and SigB regulons is increased in the Δ*aa*_3_ mutant strain of *M. smegmatis* compared to that in the WT strain, which is attributable to both the increased expression of *sigE* in the mutant and the SigE-dependent transcription of *sigB* (Oh et al., 2022). Together with the previous finding that the expression of *sigE* is significantly increased in the Δ*sigB* mutant relative to the WT strain (Oh et al., 2022), the results of our comparative determination of *rel* expression in the WT, Δ*sigB*, Δ*sigE*, Δ*aa*_3_, Δ*aa*_3_Δ*sigB*, and Δ*aa*_3_Δ*sigE* strains of *M. smegmatis* suggest that SigB, rather than SigE, is directly involved in the increased expression of *rel* in the Δ*aa*_3_ mutant (Figure 6). Furthermore, the expression of ribosomal protein genes was found to be restored in the Δ*aa*_3_Δ*mprA* mutant relative to the Δ*aa*_3_ mutant. Additionally, the increased expression of *rel* in the Δ*aa*_3_ mutant was found to require the MprBA TCS. Based on these results, we propose that the induction of SR by an increase in *rel* expression in the Δ*aa*_3_ mutant is mediated through the MprBA-SigE-SigB regulatory pathway (Figure 9). However, the mechanism by which the MprBA TCS is activated in response to inhibition of the respiratory ETC remains currently elusive. Further studies are required to answer this question. Since polyphosphate itself can serve as a phosphate donor for MprB, certain regulatory systems involved in regulating intracellular levels of polyphosphate could interconnect with the MprBA-SigE-SigB-Rel pathway to form a regulatory network for SR induction. Supporting this assumption, the depletion of inorganic phosphate has been demonstrated to lead to increased expression of *sigE* and *rel* in *M. tuberculosis* through the SenX3-RegX3 TCS that is responsible for inorganic phosphate sensing and is involved in regulating the expression of the *ppk1* gene encoding polyphosphate kinase (Rifat et al., 2009).

In conclusion, we propose that SR is induced in *M. smegmatis* under respiration-inhibitory conditions. This event appears to be primarily mediated by the MprBA-SigE-SigB-Rel pathway. Since respiration-inhibitory conditions encompass a wide range of factors that hinder the functionality of the respiratory ETC, various extracellular and intracellular conditions that can disrupt the functionality of the respiratory ETC are expected to be integrated and induce SR. They include conditions in which electron donors or terminal electron acceptors of the ETC are deficient such as nutrient starvation and hypoxia, conditions in which respiratory ETC components are inhibited such as ETC inhibitor treatment and ETC mutants, and conditions in which the proton motive force and membrane potential are affected so that the ETC is inhibited such as low pH, high ionic strength, and inhibition of the F_o_/F_1_ ATP synthase.

## Data availability statement

The datasets presented in this study can be found in online repositories. The names of the repository/repositories and accession number(s) can be found at: https://www.ncbi.nlm.nih.gov/geo/, GSE155251, GSE267048, GSE128412, GSE69983, and GSE59871.

## Author contributions

N-KK: Data curation, Formal analysis, Investigation, Writing – original draft. J-EB: Investigation, Writing – original draft. Y-JL: Investigation, Writing – original draft. YO: Data curation, Formal analysis, Investigation, Writing – original draft. J-IO: Conceptualization, Funding acquisition, Supervision, Writing – review & editing.

## Footnotes

^1^ http://eggnog5.embl.de/

^2^ https://david.ncifcrf.gov/tools.jsp
