# Supplementary material for: Rel-dependent decrease in the expression of ribosomal protein genes by inhibition of the respiratory electron transport chain in Mycobacterium smegmatis
Source: Front Microbiol. 2024 Aug 12;15:1448277. doi: 10.3389/fmicb.2024.1448277 (PMC11345224; doi:10.3389/fmicb.2024.1448277)
Supplement: Supplementary file 1 [file Data_Sheet_1.PDF]

Locus_Tag	Gene_Symbol	Product	Daa3/WT FC of gene expression	Daa3/WT raw p-value
MSMEG_0050	MSMEG_0050	hypothetical protein	2.435	0.000136
MSMEG_0051	MSMEG_0051	transcription factor WhiB family protein	4.446	0.008300
MSMEG_0055	MSMEG_0055	hypothetical protein	3.461	0.000158
MSMEG_0057	MSMEG_0057	hypothetical protein	2.027	0.004082
MSMEG_0058	MSMEG_0058	hypothetical protein	2.355	0.007666
MSMEG_0059	MSMEG_0059	ATPase AAA	2.023	0.009257
MSMEG_0062	MSMEG_0062	ftsk/SpoIIIE family protein	2.163	0.000415
MSMEG_0063	MSMEG_0063	PE family protein	3.021	0.000512
MSMEG_0064	MSMEG_0064	PPE family protein	2.629	0.001306
MSMEG_0065	MSMEG_0065	hypothetical protein	3.769	0.001753
MSMEG_0066	MSMEG_0066	WXG100 family type VII secretion target	2.788	0.000775
MSMEG_0076	MSMEG_0076	antigen MTB48	2.068	0.007729
MSMEG_0078	MSMEG_0078	hypothetical protein	2.228	0.017685
MSMEG_0206	MSMEG_0206	acyltransferase	2.208	0.002449
MSMEG_0266	MSMEG_0266	arginine decarboxylase	5.558	0.034880
MSMEG_0265	MSMEG_0265	uracil DNA glycosylase superfamily protein	8.966	0.024322
MSMEG_0267	MSMEG_0267	esterase	10.255	0.022200
MSMEG_0430	MSMEG_0430	ISL3-like element ISMsm4 family transposase	6.788	0.023351
MSMEG_0450	MSMEG_0450	hypothetical protein	7.545	0.028909
MSMEG_0451	MSMEG_0451	hypothetical protein	10.092	0.014719
MSMEG_0469	MSMEG_0469	PadR family transcriptional regulator	2.089	0.005346
MSMEG_0470	MSMEG_0470	para-nitrobenzyl esterase	3.000	0.000507
MSMEG_0472	MSMEG_0472	lipoprotein	4.095	0.005550
MSMEG_0493	MSMEG_0493	hypothetical protein	2.913	0.040615
MSMEG_0575	MSMEG_0575	MmpS1 protein	2.049	0.008028
MSMEG_0581	gabT	4-aminobutyrate aminotransferase	2.527	0.005430
MSMEG_0582	MSMEG_0582	succinate-semialdehyde dehydrogenase	2.618	0.002356
MSMEG_0586	MSMEG_0586	STAS domain-containing protein	3.044	0.010781
MSMEG_0601	MSMEG_0601	hypothetical protein	3.373	0.048830
MSMEG_0602	MSMEG_0602	hypothetical protein	2.077	0.008537
MSMEG_0637	MSMEG_0637	iron-sulfur binding oxidoreductase	6.387	0.045871
MSMEG_0651	MSMEG_0651	hypothetical protein	6.700	0.000319
MSMEG_0658	MSMEG_0658	polyamine ABC-transporter inner membrane subunit	2.326	0.012979
MSMEG_0660	MSMEG_0660	extracellular solute-binding protein	2.048	0.011936
MSMEG_0672	MSMEG_0672	hypothetical protein	7.908	0.023668
MSMEG_0685	MSMEG_0685	xanthine dehydrogenase family protein subunit M	8.986	0.027472
MSMEG_0686	MSMEG_0686	oxidoreductase	10.406	0.025847
MSMEG_0697	MSMEG_0697	integral membrane protein	6.091	0.030751
MSMEG_0703	MSMEG_0703	hypothetical protein	2.203	0.012159
MSMEG_0728	MSMEG_0728	hypothetical protein	3.032	0.014026
MSMEG_0773	MSMEG_0773	acetyltransferase	2.396	0.014186
MSMEG_0774	MSMEG_0774	pseudo	2.044	0.004069
MSMEG_0776	MSMEG_0776	hypothetical protein	2.016	0.015956
MSMEG_0816	MSMEG_0816	flavin-binding monooxygenase	2.387	0.001456
MSMEG_1076	MSMEG_1076	hypothetical protein	8.836	0.042405
MSMEG_1097	MSMEG_1097	glycosyl transferase family protein	7.809	0.044399
MSMEG_1112	MSMEG_1112	aconitate hydratase	6.913	0.025700
MSMEG_1114	MSMEG_1114	short chain dehydrogenase	2.449	0.002066
MSMEG_1131	MSMEG_1131	tryptophan-rich sensory protein	6.342	0.019308
MSMEG_1197	MSMEG_1197	LuxR family transcriptional regulator	2.373	0.002209
MSMEG_1201	MSMEG_1201	hypothetical protein	2.717	0.008057
MSMEG_1203	MSMEG_1203	methoxy mycolic acid synthase 1	2.995	0.014883
MSMEG_1205	MSMEG_1205	cyclopropane-fatty-acyl-phospholipid synthase	2.956	0.008345
MSMEG_1315	MSMEG_1315	transporter small conductance mechanosensitive ion channel (MscS) family protein	4.867	0.046300
MSMEG_1534	MSMEG_1534	hypothetical protein	2.518	0.002128
MSMEG_1533	MSMEG_1533	subtilase	2.736	0.002878
MSMEG_1535	MSMEG_1535	hypothetical protein	3.472	0.005500
MSMEG_1538	MSMEG_1538	hypothetical protein	4.152	0.000328
MSMEG_1539	MSMEG_1539	hypothetical protein	2.710	0.014109
MSMEG_1605	phoU	phosphate transporter regulatory protein PhoU	6.998	0.027753
MSMEG_1623	MSMEG_1623	short chain dehydrogenase	3.702	0.000044
MSMEG_1736	MSMEG_1736	glycerol-3-phosphate dehydrogenase	2.202	0.043682
MSMEG_1737	MSMEG_1737	RNA pseudouridine synthase	2.036	0.013775
MSMEG_1738	MSMEG_1738	transmembrane protein	3.317	0.011945
MSMEG_1750	MSMEG_1750	hypothetical protein	2.205	0.000964
MSMEG_1749	MSMEG_1749	monooxygenase	2.163	0.010571
MSMEG_1755	MSMEG_1755	anti-sigma factor	2.669	0.010799

Locus_Tag	Gene_Symbol	Product	Daa3/WT FC of gene expression	Daa3/WT raw p-value
MSMEG_1758	MSMEG_1758	hypothetical protein	11.261	0.017292
MSMEG_1766	MSMEG_1766	hypothetical protein	7.908	0.027868
MSMEG_1767	MSMEG_1767	hypothetical protein	8.249	0.029955
MSMEG_1768	MSMEG_1768	hypothetical protein	6.882	0.039271
MSMEG_1771	MSMEG_1771	methylase	10.561	0.028629
MSMEG_1773	MSMEG_1773	hypothetical protein	6.670	0.035398
MSMEG_1774	MSMEG_1774	hypothetical protein	12.568	0.020631
MSMEG_1775	MSMEG_1775	cytochrome P450 monooxygenase	6.503	0.024543
MSMEG_1777	MSMEG_1777	UsfY protein	11.360	0.013913
MSMEG_1778	MSMEG_1778	hypothetical protein	8.462	0.045546
MSMEG_1782	MSMEG_1782	SDR family oxidoreductase	10.319	0.025137
MSMEG_1783	MSMEG_1783	hypothetical protein	10.343	0.022324
MSMEG_1787	MSMEG_1787	RsbW protein	7.832	0.025204
MSMEG_1788	MSMEG_1788	hypothetical protein	11.549	0.032125
MSMEG_1791	MSMEG_1791	UsfY protein	2.832	0.025383
MSMEG_1792	MSMEG_1792	hypothetical protein	8.010	0.022489
MSMEG_1794	MSMEG_1794	dehydrogenase	9.452	0.014445
MSMEG_1802	MSMEG_1802	ChaB protein	8.495	0.020730
MSMEG_1870	MSMEG_1870	hypothetical protein	2.019	0.011005
MSMEG_1871	MSMEG_1871	hypothetical protein	2.497	0.000186
MSMEG_1872	MSMEG_1872	hypothetical protein	2.060	0.000849
MSMEG_1878	MSMEG_1878	S30AE family protein	4.373	0.012326
MSMEG_1879	MSMEG_1879	hypothetical protein	2.288	0.012105
MSMEG_1885	MSMEG_1885	2Fe-2S iron-sulfur cluster binding domain-containing protein	4.039	0.004437
MSMEG_1886	MSMEG_1886	Fatty acid desaturase	3.970	0.010481
MSMEG_1909	MSMEG_1909	HTH-type transcriptional regulator YnfL	3.324	0.020018
MSMEG_1910	MSMEG_1910	muconate cycloisomerase	2.039	0.027965
MSMEG_1950	MSMEG_1950	hypothetical protein	13.660	0.013710
MSMEG_1951	MSMEG_1951	hypothetical protein	11.569	0.027755
MSMEG_1960	MSMEG_1960	hypothetical protein	2.122	0.005301
MSMEG_1961	MSMEG_1961	hypothetical protein	2.324	0.002713
MSMEG_1962	MSMEG_1962	hypothetical protein	2.340	0.000717
MSMEG_1969	MSMEG_1969	MerR family transcriptional regulator	3.770	0.015144
MSMEG_1970	MSMEG_1970	sigma factor	8.353	0.007255
MSMEG_1971	MSMEG_1971	propane monooxygenase hydroxylase large subunit	3.304	0.001710
MSMEG_1975	MSMEG_1975	amidohydrolase	2.132	0.000111
MSMEG_2027	MSMEG_2027	hypothetical protein	3.095	0.006981
MSMEG_2112	MSMEG_2112	hypothetical protein	3.691	0.045419
MSMEG_2177	MSMEG_2177	fmnh2-utilizing oxygenase	2.755	0.005400
MSMEG_2261	MSMEG_2261	hypothetical protein	2.231	0.021037
MSMEG_2262	hybA	hydrogenase	2.286	0.014085
MSMEG_2263	hybC	nickel-dependent hydrogenase large subunit	2.074	0.005265
MSMEG_2273	hypF	[NiFe] hydrogenase maturation protein HypF	2.190	0.021402
MSMEG_2275	hypD	hydrogenase expression/formation protein HypD	2.597	0.017022
MSMEG_2276	hypE	hydrogenase expression/formation protein HypE	2.470	0.012939
MSMEG_2347	MSMEG_2347	phytoene dehydrogenase	7.296	0.047690
MSMEG_2376	MSMEG_2376	hypothetical protein	8.591	0.023912
MSMEG_2415	MSMEG_2415	hemerythrin HHE cation binding protein	8.317	0.030286
MSMEG_2416	MSMEG_2416	hypothetical protein	2.573	0.010346
MSMEG_2594	asnB	asparagine synthase	2.671	0.036453
MSMEG_2703	hypC	hydrogenase assembly chaperone HypC/HupF	2.024	0.007703
MSMEG_2714	MSMEG_2714	hypothetical protein	2.011	0.015847
MSMEG_2716	MSMEG_2716	hypothetical protein	2.362	0.018091
MSMEG_2718	MSMEG_2718	(Fe-S)-binding protein	2.373	0.000510
MSMEG_2719	MSMEG_2719	hydrogen:quinone oxidoreductase	2.642	0.023658
MSMEG_2791	MSMEG_2791	pyridoxamine 5'-phosphate oxidase	2.709	0.000346
MSMEG_2816	MSMEG_2816	hypothetical protein	2.888	0.022503
MSMEG_2837	MSMEG_2837	nitrate reductase NarB	5.590	0.031029
MSMEG_2905	MSMEG_2905	transcriptional regulator	2.094	0.001908
MSMEG_2958	MSMEG_2958	hypothetical protein	8.530	0.015118
MSMEG_3007	MSMEG_3007	succinate-semialdehyde dehydrogenase	2.475	0.000717
MSMEG_3022	MSMEG_3022	transglycosylase associated protein	11.643	0.034020
MSMEG_3137	MSMEG_3137	oxidoreductase	2.391	0.009196
MSMEG_3141	MSMEG_3141	hypothetical protein	9.889	0.039671
MSMEG_3229	MSMEG_3229	hypothetical protein	2.326	0.003702
MSMEG_3230	MSMEG_3230	pseudo	5.708	0.000029
MSMEG_3231	cydD	cysteine ABC transporter permease/ATP-binding protein	6.578	0.000523

Locus_Tag	Gene_Symbol	Product	Daa3/WT FC of gene expression	Daa3/WT raw p-value
MSMEG_3232	cydB	cytochrome D ubiquinol oxidase subunit II	8.125	0.000163
MSMEG_3233	MSMEG_3233	cytochrome D ubiquinol oxidase subunit 1	7.158	0.000379
MSMEG_3242	MSMEG_3242	starvation-inducible DNA-binding protein or fine tangled pili major subunit	2.083	0.000902
MSMEG_3243	MSMEG_3243	pseudo	2.031	0.002073
MSMEG_3244	MSMEG_3244	hypothetical protein	2.063	0.000934
MSMEG_3255	MSMEG_3255	DoxX subfamily protein	8.283	0.018143
MSMEG_3265	MSMEG_3265	arabitol-phosphate dehydrogenase	2.103	0.018030
MSMEG_3268	MSMEG_3268	ABC transporter permease	2.236	0.013601
MSMEG_3273	MSMEG_3273	M20/M25/M40 family metallo-hydrolase	7.048	0.028046
MSMEG_3289	MSMEG_3289	gp61 protein	11.762	0.026783
MSMEG_3304	MSMEG_3304	succinate semialdehyde dehydrogenase	5.678	0.042303
MSMEG_3329	MSMEG_3329	hypothetical protein	2.348	0.016638
MSMEG_3330	MSMEG_3330	hypothetical protein	2.314	0.005369
MSMEG_3331	MSMEG_3331	hypothetical protein	2.008	0.006090
MSMEG_3413	MSMEG_3413	pseudo	2.037	0.024461
MSMEG_3418	MSMEG_3418	hypothetical protein	5.824	0.042450
MSMEG_3419	MSMEG_3419	hypothetical protein	12.528	0.022899
MSMEG_3460	MSMEG_3460	ferric uptake regulation protein	4.205	0.032027
MSMEG_3461	katG	catalase/peroxidase HPI	3.424	0.031021
MSMEG_3536	MSMEG_3536	sugar transporter	5.310	0.028272
MSMEG_3540	MSMEG_3540	hypothetical protein	2.462	0.001718
MSMEG_3542	MSMEG_3542	hypothetical protein	2.095	0.000188
MSMEG_3543	MSMEG_3543	hypothetical protein	6.009	0.027846
MSMEG_3560	MSMEG_3560	hypothetical protein	3.894	0.045767
MSMEG_3562	pcaC	hypothetical protein	2.182	0.042393
MSMEG_3583	MSMEG_3583	monooxygenase	2.198	0.006682
MSMEG_3601	MSMEG_3601	ABC transporter permease	2.074	0.030559
MSMEG_3615	MSMEG_3615	zinc-binding alcohol dehydrogenase	3.141	0.010265
MSMEG_3616	MSMEG_3616	integral membrane protein	3.993	0.003827
MSMEG_3617	MSMEG_3617	hypothetical protein	2.087	0.019795
MSMEG_3630	MSMEG_3630	CopY family transcriptional regulator protein	2.752	0.002038
MSMEG_3676	MSMEG_3676	phenoxybenzoate dioxygenase subunit beta	2.377	0.011368
MSMEG_3678	MSMEG_3678	hypothetical protein	2.724	0.001408
MSMEG_3680	MSMEG_3680	hypothetical protein	4.282	0.004235
MSMEG_3811	MSMEG_3811	universal stress protein family protein	3.444	0.002864
MSMEG_3864	cobN	cobaltochelatase subunit CobN	2.278	0.003098
MSMEG_3865	MSMEG_3865	hypothetical protein	5.679	0.006086
MSMEG_3867	MSMEG_3867	ATPase AAA	2.009	0.004882
MSMEG_3868	MSMEG_3868	hypothetical protein	2.261	0.001237
MSMEG_3887	tatA	twin arginine translocase A	2.010	0.015461
MSMEG_3914	MSMEG_3914	beta-lactamase	2.444	0.000513
MSMEG_3915	MSMEG_3915	NAD-dependent alcohol dehydrogenase	2.313	0.009152
MSMEG_3941	MSMEG_3941	GAF family protein	2.029	0.002582
MSMEG_3981	MSMEG_3981	L-carnitine dehydratase/bile acid-inducible protein F	2.154	0.034517
MSMEG_3983	MSMEG_3983	pseudo	2.088	0.035879
MSMEG_4174	MSMEG_4174	IclR family transcriptional regulator	3.028	0.006379
MSMEG_4195	MSMEG_4195	hypothetical protein	4.509	0.026060
MSMEG_4298	panB	3-methyl-2-oxobutanoate hydroxymethyltransferase	2.118	0.045075
MSMEG_4330	MSMEG_4330	short chain dehydrogenase	2.507	0.016081
MSMEG_4351	MSMEG_4351	oxidoreductase YjgI	3.184	0.000333
MSMEG_4402	MSMEG_4402	plasmid pRiA4b ORF-3 family protein	4.708	0.000057
MSMEG_4465	MSMEG_4465	cutinase	6.874	0.002079
MSMEG_4500	MSMEG_4500	hypothetical protein	2.968	0.012709
MSMEG_4546	MSMEG_4546	pseudo	2.556	0.002247
MSMEG_4618	MSMEG_4618	isochorismatase	5.509	0.030345
MSMEG_4694	MSMEG_4694	HNH endonuclease	2.166	0.013396
MSMEG_4747	MSMEG_4747	hypothetical protein	2.906	0.023058
MSMEG_4748	MSMEG_4748	hypothetical protein	2.828	0.018555
MSMEG_4757	MSMEG_4757	fatty acid synthase	2.288	0.020877
MSMEG_4831	MSMEG_4831	TetR family transcriptional regulator	2.428	0.005800
MSMEG_4898	MSMEG_4898	hypothetical protein	2.067	0.019280
MSMEG_4900	MSMEG_4900	Pks14 protein	2.081	0.009862
MSMEG_4919	MSMEG_4919	hypothetical protein	2.410	0.028089
MSMEG_4921	mce	methylmalonyl-CoA epimerase	2.421	0.000990
MSMEG_4931	rrsB	16S ribosomal RNA	3.477	0.029023
MSMEG_4961	MSMEG_4961	hypothetical protein	3.178	0.001015
MSMEG_4993	MSMEG_4993	hypothetical protein	6.743	0.022379

Locus_Tag	Gene_Symbol	Product	Daa3/WT FC of gene expression	Daa3/WT raw p-value
MSMEG_5020	MSMEG_5020	acetoin(diacetyl) reductase	2.467	0.008932
MSMEG_5021	MSMEG_5021	alcohol dehydrogenase	2.890	0.001780
MSMEG_5078	glgC	glucose-1-phosphate adenylyltransferase	3.239	0.046608
MSMEG_5101	MSMEG_5101	hypothetical protein	2.293	0.000248
MSMEG_5167	MSMEG_5167	major facilitator superfamily protein	2.365	0.001722
MSMEG_5180	MSMEG_5180	hypothetical protein	3.493	0.022446
MSMEG_5181	MSMEG_5181	IS1634-like element IS1549 family transposase	2.624	0.041097
MSMEG_5188	MSMEG_5188	caax amino protease	4.766	0.043812
MSMEG_5189	MSMEG_5189	oxidoreductase	6.208	0.018456
MSMEG_5218	MSMEG_5218	hypothetical protein	2.305	0.035081
MSMEG_5230	MSMEG_5230	hypothetical protein	3.039	0.006376
MSMEG_5231	MSMEG_5231	hypothetical protein	2.136	0.008058
MSMEG_5245	MSMEG_5245	universal stress protein family protein	2.338	0.013301
MSMEG_5246	MSMEG_5246	hypothetical protein	2.077	0.002870
MSMEG_5300	MSMEG_5300	short-chain type dehydrogenase/reductase	2.518	0.001490
MSMEG_5301	MSMEG_5301	transcriptional regulator	2.315	0.001606
MSMEG_5302	MSMEG_5302	aerobic C4-dicarboxylate transporter	5.908	0.004181
MSMEG_5303	putP	sodium/proline symporter	2.342	0.010034
MSMEG_5308	MSMEG_5308	hypothetical protein	2.175	0.045855
MSMEG_5333	MSMEG_5333	hypothetical protein	2.069	0.035298
MSMEG_5334	MSMEG_5334	hypothetical protein	2.075	0.039420
MSMEG_5342	MSMEG_5342	hypothetical protein	8.378	0.026454
MSMEG_5343	MSMEG_5343	hypothetical protein	8.317	0.017235
MSMEG_5401	MSMEG_5401	hypothetical protein	4.963	0.037086
MSMEG_5402	MSMEG_5402	dehydrogenase DhgA	10.041	0.015351
MSMEG_5454	MSMEG_5454	choloylglycine hydrolase	2.415	0.018999
MSMEG_5455	MSMEG_5455	PE family protein	2.719	0.007888
MSMEG_5473	MSMEG_5473	transporter	3.257	0.034565
MSMEG_5482	mscL	large-conductance mechanosensitive channel	2.153	0.026502
MSMEG_5504	MSMEG_5504	hypothetical protein	2.095	0.002228
MSMEG_5505	MSMEG_5505	hypothetical protein	4.080	0.001146
MSMEG_5506	MSMEG_5506	hypothetical protein	2.837	0.019406
MSMEG_5507	MSMEG_5507	hypothetical protein	2.964	0.003998
MSMEG_5543	MSMEG_5543	hypothetical protein	8.242	0.027907
MSMEG_5549	MSMEG_5549	hypothetical protein	4.214	0.003623
MSMEG_5550	MSMEG_5550	pseudo	3.721	0.035988
MSMEG_5558	MSMEG_5558	hypothetical protein	2.828	0.015673
MSMEG_5559	MSMEG_5559	metabolite/sugar transporter	4.773	0.029073
MSMEG_5570	MSMEG_5570	ATP-dependent DNA ligase	2.141	0.028254
MSMEG_5575	MSMEG_5575	repressor	2.183	0.001541
MSMEG_5581	MSMEG_5581	hypothetical protein	2.068	0.008297
MSMEG_5582	MSMEG_5582	hypothetical protein	4.298	0.012928
MSMEG_5590	MSMEG_5590	carboxylate-amine ligase	9.834	0.013906
MSMEG_5605	MSMEG_5605	cytochrome bd ubiquinol oxidase subunit I	4.710	0.036794
MSMEG_5682	MSMEG_5682	hypothetical protein	2.455	0.007974
MSMEG_5722	MSMEG_5722	hypothetical protein	10.538	0.015376
MSMEG_5732	MSMEG_5732	monooxygenase	2.286	0.000591
MSMEG_5987	MSMEG_5987	two-component regulator	2.172	0.009622
MSMEG_5988	MSMEG_5988	hypothetical protein	2.493	0.004103
MSMEG_5989	MSMEG_5989	hypothetical protein	2.460	0.000292
MSMEG_6145	MSMEG_6145	hypothetical protein	2.054	0.007052
MSMEG_6210	MSMEG_6210	hypothetical protein	5.454	0.015381
MSMEG_6213	MSMEG_6213	manganese containing catalase	7.929	0.034693
MSMEG_6233	MSMEG_6233	hypothetical protein	5.799	0.037637
MSMEG_6273	MSMEG_6273	integral membrane protein	2.267	0.047563
MSMEG_6291	MSMEG_6291	D-amino-acid dehydrogenase	2.363	0.001280
MSMEG_6292	MSMEG_6292	transcription elongation factor GreA	2.134	0.024971
MSMEG_6305	MSMEG_6305	hypothetical protein	10.614	0.014679
MSMEG_6322	MSMEG_6322	bifunctional wax ester synthase/acyl-CoA diacylglycerol acyltransferase	4.083	0.043413
MSMEG_6345	MSMEG_6345	hypothetical protein	4.179	0.009222
MSMEG_6346	MSMEG_6346	hypothetical protein	3.195	0.007563
MSMEG_6347	MSMEG_6347	hypothetical protein	2.386	0.001834
MSMEG_6354	MSMEG_6354	cutinase family protein	6.290	0.025557
MSMEG_6381	MSMEG_6381	hypothetical protein	2.423	0.010405
MSMEG_6445	MSMEG_6445	hypothetical protein	2.055	0.047763
MSMEG_6446	MSMEG_6446	hypothetical protein	2.979	0.003692
MSMEG_6447	MSMEG_6447	hypothetical protein	3.161	0.008005

Locus_Tag	Gene_Symbol	Product	Daa3/WT FC of gene expression	Daa3/WT raw p-value
MSMEG_6448	MSMEG_6448	hypothetical protein	**2.519**	**0.015127**
MSMEG_6456	MSMEG_6456	hypothetical protein	**3.611**	**0.005274**
MSMEG_6467	MSMEG_6467	starvation-induced DNA protecting protein	**9.472**	**0.034992**
MSMEG_6472	MSMEG_6472	hypothetical protein	**2.536**	**0.014396**
MSMEG_6498	MSMEG_6498	hypothetical protein	**4.432**	**0.000196**
MSMEG_6500	MSMEG_6500	hypothetical protein	**3.398**	**0.047597**
MSMEG_6540	MSMEG_6540	virulence factor Mce family protein	**2.148**	**0.008001**
MSMEG_6544	MSMEG_6544	transporter	**2.116**	**0.044909**
MSMEG_6566	MSMEG_6566	hypothetical protein	**3.429**	**0.012792**
MSMEG_6610	MSMEG_6610	hypothetical protein	**6.204**	**0.049451**
MSMEG_6612	moxR	AAA domain-containing protein	**8.673**	**0.026772**
MSMEG_6615	MSMEG_6615	hypothetical protein	**8.439**	**0.049628**
MSMEG_6616	MSMEG_6616	S-(hydroxymethyl)glutathione dehydrogenase	**9.422**	**0.026488**
MSMEG_6665	MSMEG_6665	integral membrane protein	**9.578**	**0.016939**
MSMEG_6666	MSMEG_6666	hypothetical protein	**2.761**	**0.005757**
MSMEG_6667	MSMEG_6667	hypothetical protein	**7.945**	**0.035646**
MSMEG_6676	MSMEG_6676	transmembrane protein	**3.616**	**0.040973**
MSMEG_6678	MSMEG_6678	hypothetical protein	**2.260**	**0.018330**
MSMEG_6747	MSMEG_6747	oxidoreductase	**2.117**	**0.005993**
MSMEG_6768	MSMEG_6768	halogenase	**8.798**	**0.030099**
MSMEG_6801	MSMEG_6801	kinase	**2.245**	**0.013364**
MSMEG_6802	MSMEG_6802	ABC transporter ATP-binding protein	**2.357**	**0.026726**
MSMEG_6803	MSMEG_6803	ribose transporter permease RbsC	**2.479**	**0.018435**
MSMEG_6804	MSMEG_6804	sugar ABC transporter substrate-binding protein	**2.475**	**0.030822**
MSMEG_6811	MSMEG_6811	hypothetical protein	**2.021**	**0.005797**
MSMEG_6812	MSMEG_6812	pseudo	**4.605**	**0.032922**
MSMEG_6819	MSMEG_6819	pseudo	**7.792**	**0.017161**
MSMEG_6931	MSMEG_6931	RNA polymerase sigma-70 factor	**2.891**	**0.000416**

Locus_Tag	Gene_Symbol	Product	Daa3/WT FC of gene expression	Daa3/WT raw p-value
MSMEG_0132	MSMEG_0132	hypothetical protein	-2.358	0.003335
MSMEG_0133	MSMEG_0133	ABC transporter	-2.230	0.006428
MSMEG_0134	MSMEG_0134	virulence factor Mce family protein	-2.007	0.002080
MSMEG_0135	MSMEG_0135	virulence factor Mce family protein	-2.049	0.007008
MSMEG_0136	MSMEG_0136	virulence factor Mce family protein	-2.034	0.004690
MSMEG_0137	MSMEG_0137	virulence factor mce family protein	-2.038	0.014917
MSMEG_0150	MSMEG_0150	NAD(P) transhydrogenase subunit beta	-2.097	0.024763
MSMEG_0158	MSMEG_0158	formyl-coenzyme A transferase	-2.842	0.005140
MSMEG_0239	MSMEG_0239	O-acetylhomoserine/O-acetylserine sulfhydrylase	-2.163	0.013991
MSMEG_0310	MSMEG_0310	pseudo	-2.060	0.008206
MSMEG_0371	MSMEG_0371	MaoC like domain-containing protein	-2.574	0.001991
MSMEG_0372	fabG	3-ketoacyl-ACP reductase	-3.093	0.003237
MSMEG_0373	MSMEG_0373	acetyl-CoA acetyltransferase	-2.548	0.015365
MSMEG_0395	MSMEG_0395	hypothetical protein	-2.783	0.011101
MSMEG_0417	MSMEG_0417	fumarate reductase iron-sulfur subunit	-3.051	0.004932
MSMEG_0418	sdhA	succinate dehydrogenase flavoprotein subunit	-3.093	0.000514
MSMEG_0419	MSMEG_0419	integral membrane protein	-2.806	0.013605
MSMEG_0420	MSMEG_0420	hypothetical protein	-3.349	0.010473
MSMEG_0443	MSMEG_0443	carbon-nitrogen hydrolase	-2.567	0.001856
MSMEG_0444	MSMEG_0444	agmatine deiminase	-2.530	0.003337
MSMEG_0531	MSMEG_0531	acyl-CoA dehydrogenase	-2.714	0.008639
MSMEG_0532	MSMEG_0532	TetR family transcriptional regulator	-2.146	0.003395
MSMEG_0553	MSMEG_0553	sugar ABC transporter substrate-binding protein	-2.199	0.003176
MSMEG_0554	MSMEG_0554	ABC transporter permease	-2.117	0.006918
MSMEG_0627	MSMEG_0627	glycosyl transferase family protein	-2.679	0.008674
MSMEG_0644	MSMEG_0644	cupin	-2.040	0.001360
MSMEG_0694	MSMEG_0694	hypothetical protein	-2.436	0.016215
MSMEG_0781	MSMEG_0781	amino acid permease	-4.013	0.034623
MSMEG_0894	MSMEG_0894	dihydrodipicolinate reductase	-2.044	0.023044
MSMEG_0911	aceA	isocitrate lyase	-2.391	0.009104
MSMEG_0914	MSMEG_0914	hypothetical protein	-2.374	0.000233
MSMEG_0961	MSMEG_0961	DUF2236 domain-containing protein	-2.096	0.005355
MSMEG_0965	MSMEG_0965	porin	-2.000	0.030510
MSMEG_0966	MSMEG_0966	hypothetical protein	-2.066	0.014170
MSMEG_1171	MSMEG_1171	low molecular weight phosphotyrosine protein phosphatase	-3.333	0.013433
MSMEG_1172	arsB	arsenic resistance protein	-3.924	0.024430
MSMEG_1173	MSMEG_1173	ArsR family transcriptional regulator	-4.298	0.017341
MSMEG_1176	MSMEG_1176	hypothetical protein	-2.321	0.007417
MSMEG_1202	MSMEG_1202	hypothetical protein	-2.489	0.019589
MSMEG_1212	MSMEG_1212	hypothetical protein	-3.235	0.029828
MSMEG_1254	MSMEG_1254	DEAD/DEAH box helicase	-2.067	0.024521
MSMEG_1255	MSMEG_1255	UvrD/REP helicase	-3.972	0.000264
MSMEG_1256	MSMEG_1256	hypothetical protein	-3.558	0.000733
MSMEG_1257	MSMEG_1257	plasmid pRiA4b ORF-3 family protein	-2.119	0.006500
MSMEG_1264	MSMEG_1264	prophage Lp1 protein 5	-2.106	0.043218
MSMEG_1316	prfC	peptide chain release factor 3	-2.037	0.020202
MSMEG_1330	MSMEG_1330	MarR family transcriptional regulator	-2.020	0.003601
MSMEG_1338	MSMEG_1338	tRNA-Met	-2.215	0.037667
MSMEG_1340	MSMEG_1340	(3R)-hydroxyacyl-ACP dehydratase subunit HadA	-2.337	0.013071
MSMEG_1347	rplA	50S ribosomal protein L1	-2.062	0.019451
MSMEG_1348	MSMEG_1348	RNA polymerase ECF-subfamily protein sigma factor	-2.183	0.008211
MSMEG_1349	MSMEG_1349	DGPF domain-containing protein	-3.005	0.003086
MSMEG_1364	rplJ	50S ribosomal protein L10	-2.201	0.042699
MSMEG_1365	rplL	50S ribosomal protein L7/L12	-2.434	0.008811
MSMEG_1420	MSMEG_1420	transcriptional regulatory protein	-2.358	0.027249
MSMEG_1421	MSMEG_1421	hypothetical protein	-5.840	0.015199

Locus_Tag	Gene_Symbol	Product	Daa3/WT FC of gene expression	Daa3/WT raw p-value
MSMEG_1422	MSMEG_1422	hypothetical protein	-5.690	0.012858
MSMEG_1424	MSMEG_1424	FMN-dependent dehydrogenase	-4.783	0.041532
MSMEG_1429	MSMEG_1429	cytochrome P450-terp	-2.149	0.003670
MSMEG_1436	rplC	50S ribosomal protein L3	-2.108	0.029327
MSMEG_1437	rplD	50S ribosomal protein L4	-2.047	0.021867
MSMEG_1440	rpsS	30S ribosomal protein S19	-2.017	0.026858
MSMEG_1441	rplV	50S ribosomal protein L22	-2.027	0.037431
MSMEG_1442	rpsC	30S ribosomal protein S3	-2.138	0.017395
MSMEG_1444	rpmC	50S ribosomal protein L29	-2.433	0.031336
MSMEG_1445	MSMEG_1445	30S ribosomal protein S17	-2.084	0.037443
MSMEG_1446	MSMEG_1446	NTP pyrophosphohydrolase	-2.981	0.010963
MSMEG_1447	MSMEG_1447	MerR family transcriptional regulator	-3.837	0.043609
MSMEG_1448	MSMEG_1448	integral membrane transporter	-4.312	0.037224
MSMEG_1465	rplN	50S ribosomal protein L14	-2.032	0.028024
MSMEG_1466	rplX	50S ribosomal protein L24	-2.178	0.012836
MSMEG_1467	rplE	50S ribosomal protein L5	-2.060	0.018701
MSMEG_1469	rpsH	30S ribosomal protein S8	-2.189	0.013062
MSMEG_1470	rplF	50S ribosomal protein L6	-2.187	0.028845
MSMEG_1471	rplR	50S ribosomal protein L18	-2.222	0.035421
MSMEG_1472	rpsE	30S ribosomal protein S5	-2.138	0.022395
MSMEG_1473	rpmD	50S ribosomal protein L30	-2.250	0.040435
MSMEG_1474	rplO	50S ribosomal protein L15	-2.340	0.006731
MSMEG_1525	MSMEG_1525	50S ribosomal protein L17	-2.122	0.012944
MSMEG_1559	glmM	phosphoglucosamine mutase	-2.199	0.000953
MSMEG_1628	MSMEG_1628	SDR family NAD(P)-dependent oxidoreductase	-2.738	0.000826
MSMEG_1632	MSMEG_1632	hypothetical protein	-2.323	0.001546
MSMEG_1634	MSMEG_1634	forkhead-associated protein	-3.434	0.006963
MSMEG_1711	MSMEG_1711	ABC transporter ATP-binding protein	-3.050	0.002511
MSMEG_1712	MSMEG_1712	ABC transporter periplasmic protein	-2.458	0.013399
MSMEG_1762	MSMEG_1762	piperideine-6-carboxylic acid dehydrogenase	-3.846	0.031095
MSMEG_1764	MSMEG_1764	L-lysine aminotransferase	-12.085	0.001357
MSMEG_1765	MSMEG_1765	restriction endonuclease	-4.085	0.034095
MSMEG_1880	MSMEG_1880	hypothetical protein	-2.797	0.011905
MSMEG_1965	MSMEG_1965	tRNA-Met	-3.768	0.046344
MSMEG_2058	nuoF	NADH-quinone oxidoreductase subunit F	-3.151	0.045713
MSMEG_2080	MSMEG_2080	acyl-CoA dehydrogenase	-2.044	0.016871
MSMEG_2081	MSMEG_2081	acyl-CoA dehydrogenase	-2.179	0.022051
MSMEG_2116	MSMEG_2116	PTS transporter subunit EIIC	-2.159	0.001645
MSMEG_2221	MSMEG_2221	hypothetical protein	-2.302	0.028177
MSMEG_2257	MSMEG_2257	cytochrome P450-terp	-2.503	0.001880
MSMEG_2380	MSMEG_2380	sugar transporter family protein	-2.091	0.015208
MSMEG_2381	MSMEG_2381	hypothetical protein	-2.151	0.018548
MSMEG_2385	MSMEG_2385	tRNA-Glu	-2.085	0.010319
MSMEG_2425	amt	ammonium transporter	-2.624	0.010263
MSMEG_2435	rpsP	30S ribosomal protein S16	-2.157	0.004937
MSMEG_2436	MSMEG_2436	hypothetical protein	-2.372	0.004369
MSMEG_2437	rimM	16S rRNA-processing protein RimM	-3.785	0.003055
MSMEG_2438	trmD	tRNA (guanine-N(1)-)-methyltransferase	-3.066	0.010115
MSMEG_2474	MSMEG_2474	cutinase Cut3	-2.106	0.019640
MSMEG_2475	MSMEG_2475	oxidoreductase YdbC	-2.665	0.001498
MSMEG_2520	tsf	elongation factor Ts	-2.110	0.021108
MSMEG_2527	MSMEG_2527	hypothetical protein	-2.034	0.007569
MSMEG_2551	MSMEG_2551	hypothetical protein	-2.340	0.019802
MSMEG_2574	MSMEG_2574	hypothetical protein	-2.054	0.001400
MSMEG_2634	MSMEG_2634	hypothetical protein	-2.264	0.003306
MSMEG_2654	rpsO	30S ribosomal protein S15	-2.433	0.020891

Locus_Tag	Gene_Symbol	Product	Daa3/WT FC of gene expression	Daa3/WT raw p-value
MSMEG_2655	MSMEG_2655	LppU protein	-3.802	0.004252
MSMEG_2799	MSMEG_2799	phospho-2-dehydro-3-deoxyheptonate aldolase	-2.262	0.013830
MSMEG_2798	MSMEG_2798	hypothetical protein	-2.081	0.004866
MSMEG_2840	MSMEG_2840	hypothetical protein	-2.099	0.007006
MSMEG_2844	MSMEG_2844	ABC transporter ATP-binding protein	-2.036	0.006687
MSMEG_2899	MSMEG_2899	short chain dehydrogenase	-2.165	0.022650
MSMEG_2900	MSMEG_2900	alpha/beta fold hydrolase	-2.155	0.002705
MSMEG_2977	MSMEG_2977	hypothetical protein	-2.726	0.007729
MSMEG_2982	MSMEG_2982	periplasmic binding protein	-2.128	0.005094
MSMEG_2984	MSMEG_2984	hydrolase	-2.150	0.004465
MSMEG_3058	MSMEG_3058	metal ABC transporter substrate-binding protein	-2.387	0.018187
MSMEG_3152	hemH	ferrochelatase	-2.035	0.001478
MSMEG_3297	MSMEG_3297	CadC family transcriptional regulator	-3.864	0.004910
MSMEG_3298	MSMEG_3298	response regulator receiver domain-containing protein	-4.265	0.005970
MSMEG_3299	MSMEG_3299	pseudo	-2.396	0.029477
MSMEG_3301	MSMEG_3301	hypothetical protein	-2.353	0.007646
MSMEG_3302	MSMEG_3302	short-chain dehydrogenase/reductase SDR	-2.073	0.005225
MSMEG_3337	MSMEG_3337	hypothetical protein	-2.124	0.036854
MSMEG_3363	MSMEG_3363	TetR family transcriptional regulator	-2.248	0.010192
MSMEG_3364	MSMEG_3364	RhtB family protein transporter	-2.077	0.004313
MSMEG_3379	MSMEG_3379	2-deoxy-D-gluconate 3-dehydrogenase	-2.128	0.012227
MSMEG_3451	MSMEG_3451	short-chain dehydrogenase/reductase	-2.166	0.000077
MSMEG_3484	MSMEG_3484	cupin	-2.347	0.002675
MSMEG_3499	MSMEG_3499	hypothetical protein	-2.392	0.005715
MSMEG_3531	MSMEG_3531	hypothetical protein	-2.375	0.001339
MSMEG_3533	MSMEG_3533	di-/tripeptide transporter	-2.419	0.016087
MSMEG_3532	MSMEG_3532	serine/threonine dehydratase	-2.375	0.026729
MSMEG_3544	MSMEG_3544	hypothetical protein	-2.023	0.035426
MSMEG_3545	MSMEG_3545	hypothetical protein	-2.103	0.007921
MSMEG_3642	gcvP	glycine dehydrogenase	-2.292	0.006907
MSMEG_3701	MSMEG_3701	hypothetical protein	-2.456	0.013999
MSMEG_3708	MSMEG_3708	catalase	-2.052	0.042410
MSMEG_3734	MSMEG_3734	tRNA-Pro	-2.473	0.036399
MSMEG_3845	MSMEG_3845	hypothetical protein	-2.161	0.034165
MSMEG_3898	thpD	ectoine hydroxylase	-2.677	0.008362
MSMEG_3899	ectC	L-ectoine synthase	-3.767	0.001678
MSMEG_3900	ectB	diaminobutyrate--2-oxoglutarate aminotransferase	-3.597	0.002328
MSMEG_3997	MSMEG_3997	regulatory protein	-2.744	0.001171
MSMEG_4063	MSMEG_4063	amidohydrolase	-2.190	0.003826
MSMEG_4078	MSMEG_4078	metabolite transporter YaaU	-2.323	0.004487
MSMEG_4082	MSMEG_4082	monoxygenase	-2.484	0.001362
MSMEG_4083	MSMEG_4083	monooxygenase	-2.623	0.003402
MSMEG_4084	MSMEG_4084	acyl-CoA dehydrogenase	-2.438	0.006834
MSMEG_4085	MSMEG_4085	nitrilotriacetate monooxygenase component A	-2.751	0.003076
MSMEG_4086	ssuD	nitrilotriacetate monooxygenase component A	-2.907	0.000280
MSMEG_4087	MSMEG_4087	major facilitator superfamily protein	-2.869	0.001582
MSMEG_4094	MSMEG_4094	acyl-CoA dehydrogenase	-2.215	0.000315
MSMEG_4224	MSMEG_4224	hypothetical protein	-2.563	0.007503
MSMEG_4424	MSMEG_4424	endoribonuclease L-PSP	-2.642	0.010958
MSMEG_4426	MSMEG_4426	hypothetical protein	-2.160	0.015454
MSMEG_4478	MSMEG_4478	tRNA-Asn	-2.412	0.004777
MSMEG_4623	obgE	GTPase ObgE	-2.559	0.014859
MSMEG_4627	ndk	nucleoside diphosphate kinase	-2.278	0.003596
MSMEG_4726	MSMEG_4726	carbamoyl-phosphate synthase small subunit	-2.040	0.015906
MSMEG_4727	MSMEG_4727	mycocerosic acid synthase	-5.270	0.036026
MSMEG_4728	MSMEG_4728	condensation domain-containing protein	-6.145	0.013553

Locus_Tag	Gene_Symbol	Product	Daa3/WT FC of gene expression	Daa3/WT raw p-value
MSMEG_4729	MSMEG_4729	hypothetical protein	-5.524	0.027021
MSMEG_4730	MSMEG_4730	hypothetical protein	-6.381	0.012072
MSMEG_4731	MSMEG_4731	acyl-CoA synthetase	-6.516	0.030824
MSMEG_4732	MSMEG_4732	glycosyl transferase family protein	-6.778	0.013575
MSMEG_4733	MSMEG_4733	hypothetical protein	-6.131	0.008279
MSMEG_4734	MSMEG_4734	hypothetical protein	-5.395	0.001176
MSMEG_4735	MSMEG_4735	hypothetical protein	-5.584	0.001178
MSMEG_4736	MSMEG_4736	hypothetical protein	-3.541	0.008668
MSMEG_4737	MSMEG_4737	hypothetical protein	-3.864	0.014006
MSMEG_4738	MSMEG_4738	hypothetical protein	-4.062	0.016460
MSMEG_4739	MSMEG_4739	hypothetical protein	-3.138	0.000803
MSMEG_4740	MSMEG_4740	glycosyltransferase 28	-3.453	0.019135
MSMEG_4741	MSMEG_4741	MmpL protein	-2.783	0.005022
MSMEG_4750	MSMEG_4750	ornithine carbamoyltransferase	-2.327	0.011873
MSMEG_4848	MSMEG_4848	hypothetical protein	-2.006	0.028976
MSMEG_4892	MSMEG_4892	hypothetical protein	-2.038	0.008847
MSMEG_4928	MSMEG_4928	methylated-DNA--protein-cysteine methyltransferase	-2.928	0.042905
MSMEG_5105	MSMEG_5105	hypothetical protein	-2.189	0.002175
MSMEG_5294	MSMEG_5294	hypothetical protein	-2.314	0.012562
MSMEG_5322	MSMEG_5322	hypothetical protein	-5.335	0.035855
MSMEG_5369	ehuC	ectoine/hydroxyectoine ABC transporter permease EhuC	-2.125	0.002037
MSMEG_5403	cadA	cadmium-translocating P-type ATPase	-5.458	0.004104
MSMEG_5432	pth	peptidyl-tRNA hydrolase	-3.211	0.022183
MSMEG_5434	MSMEG_5434	hypothetical protein	-2.314	0.007728
MSMEG_5533	MSMEG_5533	4Fe-4S ferredoxin	-2.680	0.004341
MSMEG_5548	cobF	precorrin 6A synthase	-2.255	0.000851
MSMEG_5557	MSMEG_5557	hypothetical protein	-2.180	0.017544
MSMEG_5651	MSMEG_5651	LuxR family transcriptional regulator	-2.175	0.004092
MSMEG_5755	MSMEG_5755	tRNA-Phe	-3.696	0.000202
MSMEG_5942	MSMEG_5942	AMP-dependent synthetase/ligase	-2.041	0.000311
MSMEG_6116	MSMEG_6116	OHCU decarboxylase	-2.678	0.002570
MSMEG_6115	MSMEG_6115	phosphoglycerate dehydrogenase	-2.097	0.028867
MSMEG_6156	MSMEG_6156	plasmid pRiA4b ORF-3 family protein	-2.607	0.002002
MSMEG_6236	MSMEG_6236	response regulator transcription factor	-2.468	0.044694
MSMEG_6239	MSMEG_6239	iron-containing alcohol dehydrogenase	-2.180	0.004719
MSMEG_6240	MSMEG_6240	hypothetical protein	-4.717	0.017860
MSMEG_6241	MSMEG_6241	ATPase AAA	-4.852	0.024920
MSMEG_6242	MSMEG_6242	alcohol dehydrogenase	-3.691	0.013532
MSMEG_6313	tgt	queuine tRNA-ribosyltransferase	-2.170	0.007035
MSMEG_6315	MSMEG_6315	lipoprotein LpqH	-4.170	0.001226
MSMEG_6314	MSMEG_6314	haloalkane dehalogenase	-3.250	0.003330
MSMEG_6383	MSMEG_6383	transcription regulator FurA	-3.262	0.003941
MSMEG_6384	katG	catalase/peroxidase HPI	-4.258	0.001155
MSMEG_6390	MSMEG_6390	transporter major facilitator family protein	-3.409	0.000690
MSMEG_6391	MSMEG_6391	propionyl-CoA carboxylase subunit beta	-2.020	0.019347
MSMEG_6392	MSMEG_6392	polyketide synthase	-2.204	0.011021
MSMEG_6393	MSMEG_6393	long-chain-fatty-acid--CoA ligase	-2.245	0.005735
MSMEG_6436	MSMEG_6436	copper resistance protein CopC	-2.310	0.003227
MSMEG_6437	MSMEG_6437	copper resistance protein D	-2.146	0.007727
MSMEG_6458	gltD	glutamate synthase subunit beta	-2.695	0.021145
MSMEG_6459	MSMEG_6459	ferredoxin-dependent glutamate synthase 1	-2.355	0.034620
MSMEG_6519	MSMEG_6519	pyridoxamine 5'-phosphate oxidase	-2.123	0.002958
MSMEG_6523	MSMEG_6523	ABC transporter	-2.009	0.011472
MSMEG_6584	MSMEG_6584	acyl-CoA dehydrogenase	-2.163	0.002446
MSMEG_6586	MSMEG_6586	alpha/beta hydrolase	-2.112	0.006909
MSMEG_6649	MSMEG_6649	hypothetical protein	-2.693	0.000725

Locus_Tag	Gene_Symbol	Product	Daa3/WT FC of gene expression	Daa3/WT raw p-value
MSMEG_6743	MSMEG_6743	lactoylglutathione lyase	**-2.058**	**0.018618**
MSMEG_6752	MSMEG_6752	endoglucanase A	**-2.188**	**0.000105**
MSMEG_6754	MSMEG_6754	(R)-hydratase	**-2.562**	**0.004963**
MSMEG_6763	MSMEG_6763	oxidoreductase	**-2.138**	**0.000244**
MSMEG_6894	rplI	50S ribosomal protein L9	**-2.375**	**0.005289**
MSMEG_6895	rpsR	30S ribosomal protein S18	**-2.342**	**0.024743**
MSMEG_6896	MSMEG_6896	single-stranded DNA-binding protein	**-2.280**	**0.005878**
MSMEG_6897	rpsF	30S ribosomal protein S6	**-2.227**	**0.013901**
MSMEG_6945	rnpA	ribonuclease P protein component	**-2.096**	**0.002708**
